# Longitudinal change and prognostic significance of serum PINK1 levels, and mediation role of delayed cerebral ischemia in human aneurysmal subarachnoid hemorrhage: an observational analytical study

**DOI:** 10.3389/fneur.2025.1601855

**Published:** 2025-09-05

**Authors:** Guo-Jun He, Feng-Ju Xie, Fei-Long He, Wei-Fang Ni, Si-Hua Chen, Gang Wang

**Affiliations:** ^1^Department of Neurosurgery, The Affiliated Hospital of Shaoxing University (Shaoxing Municipal Hospital), Shaoxing, China; ^2^Department of Anesthesiology, The Affiliated Hospital of Shaoxing University (Shaoxing Municipal Hospital), Shaoxing, China

**Keywords:** aneurysm, subarachnoid hemorrhage, prognosis, severity, PTEN-induced putative kinase 1, delayed cerebral ischemia

## Abstract

**Background:**

PTEN-induced putative kinase 1 (PINK1) is an endogenous protective protein. This study analyzed the prognostic implications of serum PINK1 levels and assessed the mediation effect of delayed cerebral ischemia (DCI) in human aneurysmal subarachnoid hemorrhage (aSAH).

**Methods:**

In this observational analytical study of 201 aSAH patients and 87 healthy controls, serum PINK1 levels were measured at admission of all patients, from admission up to day 14 in 87 of all patients and at study entry in controls. The modified Fisher scale (mFisher) and World Federation of Neurological Surgeons scale (WFNS) were recorded for reflecting severity, and in-hospital DCI and post-aSAH 90-day Glasgow Outcome Scale (GOS) were documented as the two outcome variables.

**Results:**

Serum PINK1 levels of patients were sharply increased at admission, and were still higher until day 14 than those of controls. Admission serum PINK1 levels were independently correlated with WFNS scores and mFisher scores, were linearly related to probabilities of DCI and poor prognosis, were independently associated with continuous GOS scores, ordinal GOS scores, DCI, and poor prognosis, and efficiently distinguished risks of DCI and poor prognosis under the receiver operating characteristic curve. The models of DCI and poor prognosis, encompassing their respective independent predictors, performed well. The association between admission serum PINK1 level and poor prognosis was partially mediated by DCI.

**Conclusion:**

A notable elevation in serum PINK1 levels after aSAH is strongly related to illness severity, worse 90-day prognosis, and DCI, and DCI may in part navigate the links between serum PINK1 levels and poor prognosis following aSAH.

## 1 Introduction

Aneurysmal subarachnoid hemorrhage (aSAH) is one of the most common causes and can lead to high mortality and disability rate in the neurologic field globally (1). Although advanced techniques have been rapidly developed to satisfactorily secure aneurysms, effective drugs or strategies have not yet been discovered for restoring neuronal injury (2). Early brain injury and delayed cerebral ischemia (DCI) are the two pivotal pathophysiological mechanisms, which are involved in secondary brain injury after aSAH (3, 4). The complex molecular events cover inflammation, oxidation, apoptosis and so on (5, 6). DCI is a common adverse event in clinical practice following aSAH, and can easily make patients at risk of poor prognosis (7). The World Federation of Neurosurgical Societies (WFNS) and modified Fisher (mFisher) scales are favorably adopted by neurosurgeons for severity assessment and prognosis prediction in aSAH (8, 9). The Glasgow Outcome Scale (GOS) is widely used to evaluate neurological outcome of aSAH (10). Apart from diagnosis and therapy, prognostic analysis is particularly important for aSAH managements (1, 2). Notably, owing to their speedy and convenient accessibility, blood markers have been extensively noted with respect to their prognostic implications in aSAH in recent decades (11–13).

Autophagy is an intracellular self-repair system. It functionally maintains cellular homeostasis, energy balance, and stable cell signaling, and subsequently restores cellular functions and ensures cellular survival (14–16). PTEN-induced putative kinase 1 (PINK1) is a mitochondrial-targeted serine/threonine-protein kinase, harbor endogenous protective function, and participates in mitochondrial autophagy (17). PINK1 could protect brain tissues against traumatic, ischemic, and hemorrhagic insults (18–20). PINK1 expressions were significantly enhanced in cerebral cortices of rats following SAH (21). Alternatively, brain PINK1 mRNA expressions were substantially up-regulated in patients with intracerebral hemorrhage, and the increased levels were strongly correlated with hemorrhagic amount (22). Moreover, PINK1 levels in peripheral blood and cerebrospinal fluid of patients with multiple sclerosis or Alzheimer’s disease were markedly heightened, as compared to controls (23, 24). Also, in a clinical study of 56 patients with aSAH, PINK1 mRNA expression levels were notably elevated in cerebrospinal fluid of patients with DCI, relative to the other remainders (25, 26). Thus, PINK1 may be a feasible biomarker of brain injury. This study aimed to determine the temporal trends of serum PINK1 levels, the predictive effects of serum PINK1 on DCI and poor prognosis, and the mediating role of DCI in a cohort of patients with aSAH.

## 2 Methods and materials

### 2.1 Study design and plan

This observational analytical study was conducted at the Affiliated Hospital of Shaoxing University (Shaoxing, China) between January 2019 and January 2024. All patients with aSAH and the controls were recruited according to the criteria shown in Supplementary Figure 1. This study was divided into two parts: the cross-sectional study and the prospective cohort study (Supplementary Figure 2). The former was designed to discern the evolutionary trajectory of serum PINK1 levels following aSAH by using controls and a portion of patients with aSAH consenting for supplying serial samples and the latter was performed to ascertain the prognostic value of serum PINK1 levels by applying all patients with aSAH. This study adhered to the relevant institutional, local, and national laws, and obeyed the tenets of the Declaration of Helsinki and its subsequent updates. The study protocol was agreed by the Institutional Ethics Review Committee at the Affiliated Hospital of Shaoxing University (Approval Number: SMH202511001). Controls and patients’ lawful proxies were notified of study details and independently signed informed consent forms.

### 2.2 Data collection

Conventional information, such as demographic data, lifestyle habits, medical history, medication history, and admission vital signs were documented upon arrival at the emergency center. The initial WFNS and mFisher scores were recorded to evaluate severity. Hemorrhagic extension into the intraventricular system and acute hydrocephalus were judged via head computed tomography imaging. The intracranial aneurysms were observed to identify their location, size, and shape. Aneurysm size was measured on a 0.1-mm scale using electronic calipers on computed tomography angiography scans or digital subtraction angiography scans. Aneurysm location and morphology were identified by visual assessment. Aneurysms were secured using neurosurgical clipping or endovascular intervention. External ventricular drainage was performed as necessary. DCI was diagnosed according to previously published standards: (1) clinical deterioration (i.e., new focal deficits, decreased level of consciousness, or both), and/or (2) new infarction on head computed tomography scan at admission or immediately postoperatively that could not be attributed to other causes by clinical assessment, brain imaging, or appropriate laboratory studies (27). Via telephone visits at 90 days after aSAH, GOS scores were assessed through structured interviews by a trained technician; and the scores of 1–3 indicated poor prognosis (28).

### 2.3 Serum PINK1 measurements

According to the voluntary compliance principle, venous blood was drawn at admission from all patients and, at admission and at days 1, 3, 5, 7, 10, and 14 after aSAH from a portion of patients. Blood samples of controls were collected at their study entry. Blood specimens were promptly placed into gel-prefilled tubes, and centrifuged at 2,000 × g for 10 min, and then the supernatants were transferred to Eppendorf tubes and stored at −80 °C until subsequent measurements. To prevent protein breakdown, the time from blood sampling to biomarker measurement was limited within 3 months. By applying the Enzyme-Linked Immunosorbent Assay kit (Wuhan Feien Biotechnology Co., Ltd., Wuhan, China; Article No. EH15183), serum PINK1 levels were quantified in duplicate by the same professional personnel who were blinded to the clinical materials. For this kit, detection range was from 0.156 to 10 ng/mL, sensitivity was 0.094 ng/mL, and both intra- and inter-assay coefficients of variation were below 10%.

### 2.4 Statistical analysis

SPSS 25.0 (BMI Software Inc., United States) was used for statistical analysis. Categorical variables were summarized as counts (proportions). Following the Shapiro–Wilk test or Kolmogorov–Smirnov test as applicable, continuous variables were reported as means (standard deviations, SDs) if normally distributed, and as the median (25th–75th percentiles) if non-normally distributed. Data were compared between the two groups by applying the chi-square test, Fisher’s exact test, Mann–Whitney *U* test or independent *t*-test as appropriate, and data among several groups were compared using the Kruskal–Wallis test. Bivariate correlations were assessed using the Spearman’s test. Multivariate analytical approaches encompassed multivariate linear regression, binary logistic regression, and ordinal regression analyses; and the dependent variables included serum PINK1 levels, continuous GOS scores, binary GOS scores (poor prognosis versus good prognosis), DCI and ordinal GOS scores. In the framework of R software (version 4.2.4; http://www.r-project.org), two nomograms were drawn to visualize combined models of DCI and poor prognosis, and the calibration curve and decision curve were created to assess the stability and validity of the models. A restricted cubic spline was used to confirm the linear relationship. Mediation analysis was performed to determine the role of DCI in mediating the association between serum PINK1 levels and poor prognosis. GraphPad Prism 9.0 (GraphPad Software, Inc., Boston, MA, United States) was selected for drawing the receiver operating characteristic (ROC) curve. The area under the ROC curve (AUC) was compared using the *Z*-test by employing the MedCalc 20.305 (MedCalc Software, Mariakel, Belgium). With type 1 error at 0.05, test power at 0.95 and effect size at 0.8, sample size was estimated for comparing serum PINK1 levels; and results were validated via *a priori* power analysis in help of the G*Power 3.1.9.4 (Heinrich-Heine-Universität Düsseldorf, Universitätsstraße 1, Düsseldorf, Germany). Variance inflation factor (VIF) value was calculated for multicollinearity judgement, with the value above 10 denoting presence of multicollinearity. Statistical significance was defined as a two-tailed *p*-value <0.05.

## 3 Results

### 3.1 Participant characteristics

According to the recruitment criteria in Supplementary Figure 1, a total of 264 patients with aSAH were initially assessed, 63 patients were afterwards excluded and 201 patients were finally retained for clinical investigation. Eighty-seven among 201 patients permitted for serial samplings. Baseline features were not significantly different between all patients and those 87 patients (all *p* > 0.05; Table 1). Eighty-seven controls were aged from 33 to 76 years (mean, 52.4 years; SD, 9.5 years), included 45 females, 42 males, 21 tobacco smokers, 22 alcohol consumers, 6 diabetic patients, 17 hypertensive patients and 21 hyperlipidemic patients. The aforementioned seven parameters were not substantially different between controls and those 87 patients (all *p* > 0.05).

**Table 1:** Basic features between all patients and those consenters for multiple-time sampling after aneurysmal subarachnoid hemorrhage.

Parameters	All patients	Certain patients	*p*-value
Counts	201	87	
Gender (male/female)	91/110	40/47	0.912
Age (years)	51.7 ± 9.9	52.2 ± 10.5	0.683
Cigarette smoking	57 (28.4%)	23 (26.4%)	0.738
Alcohol consumption	61 (30.3%)	25 (28.7%)	0.784
Hyperlipidemia	54 (26.9%)	16 (18.4%)	0.124
Diabetes mellitus	14 (7.0%)	4 (4.6%)	0.446
Hypertension	39 (19.4%)	15 (17.2%)	0.666
WFNS scores	3 (2–3)	3 (2–4)	0.510
Modified Fisher scores	2 (1–3)	2 (2–3)	0.940
Aneurysmal position (posterior/anterior circulation)	56/145	23/64	0.804
Aneurysmal shape (cystic/others)	163/38	66/21	0.312
Aneurysmal diameter (<10 mm/≥10 mm)	118/83	51/36	0.989
Methods for securing aneurysms (clipping/endovascular intervention)	84/117	37/50	0.907
Acute hydrocephalus	33 (16.4%)	9 (10.3%)	0.180
Intraventricular extension of bleeding	39 (19.4%)	16 (18.4%)	0.841
External ventricular drain	26 (12.9%)	10 (11.5%)	0.734
Admission time since stroke (h)	8.1 (4.0–13.0)	8.4 (4.6–13.4)	0.394
Sampling time since stroke (h)	8.9 (4.6–13.5)	9.6 (5.7–13.8)	0.386
Systolic arterial pressure (mmHg)	138.5 ± 23.6	136.9 ± 25.9	0.623
Diastolic arterial pressure (mmHg)	87.2 ± 11.5	85.9 ± 12.1	0.390
Blood glucose levels (mmol/L)	11.4 (8.5–15.9)	11.0 (8.0–16.4)	0.676
Blood leucocyte counts (×10^9^/L)	7.7 (5.3–10.8)	7.6 (5.3–10.6)	0.739
Admission serum PINK1 levels (ng/mL)	4.8 (3.7–7.8)	4.9 (3.8–7.6)	0.869
Delayed cerebral ischemia	56 (27.9%)	25 (28.7%)	0.879
Continuous GOS scores at 90 days	4 (3–4)	4 (3–4)	0.937
Ordinal GOS scores at 90 days			0.819
1	16	6	
2	24	8	
3	37	21	
4	75	32	
5	49	20	
Poor prognosis (GOS scores of 1–3) at 90 days	77 (38.3%)	35 (40.2%)	0.759

Data were presented in form of median (25th–75th percentiles), mean (standard deviation) or frequency (percentage) as deemed suitable. Disparities were discerned in application of the *χ*^2^ test, Fisher’s exact test, *t*-test or Mann–Whitney *U*-test as considered applicable. PINK1, PTEN-induced putative kinase 1; WFNS, World Federation of Neurological Surgeons scale; GOS, Glasgow Outcome Scale.

### 3.2 Serum PINK1 levels and sickness severity

**Figure 1:**
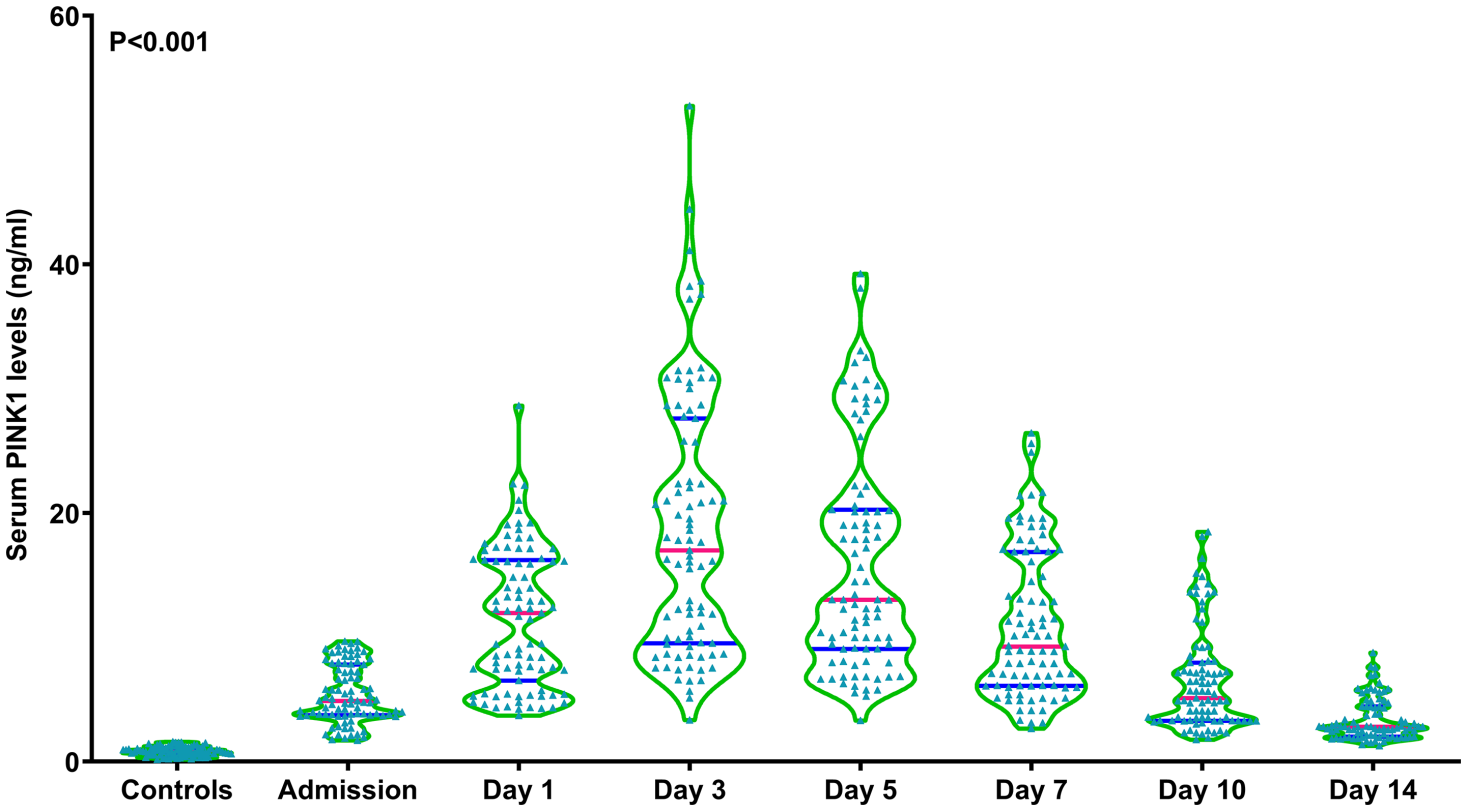
Longitudinal change of serum-based PTEN-induced putative kinase 1 levels following aneurysmal subarachnoid hemorrhage. Serum-based PTEN-induced putative kinase 1 levels rapidly increased at admission of patients with aneurysmal subarachnoid hemorrhage, remaining substantially higher until day 14 after disease onset compared to the controls, and peaking on day 3 after vascular attack (*p* < 0.001). PINK1, PTEN-induced putative kinase 1.

As shown in Figure 1, serum PINK1 levels were promptly elevated upon patient admission, gradually increased on day 1, peaked on day 3, declined from day 5 to day 14, and were markedly higher during 14 days after aSAH in patients than in controls (*p* < 0.001). Serum PINK1 levels since admission until day 14 after aSAH were markedly positively correlated with WFNS (all *p* < 0.01; Supplementary Table 1) and mFisher scores (all *p* < 0.01; Supplementary Table 2). Among all patients, serum PINK1 levels at admission were closely positively related to continuous WFNS scores (*p* < 0.001; Supplementary Figure 3) and categorical WFNS scores (*p* < 0.001; Supplementary Figure 4), as well as were strongly positively correlated with continuous mFisher scores (*p* < 0.001; Supplementary Figure 5) and categorical mFisher scores (*p* < 0.001; Supplementary Figure 6). Moreover, apart from the WFNS and mFisher scores, acute hydrocephalus and intraventricular accumulation of bleeding were strongly correlated with serum PINK1 levels at admission in all patients (all *p* < 0.05; Table 2). Following integration of the preceding four significantly correlated variables into the multivariate linear regression model, serum PINK1 levels at admission were independently correlated with WFNS scores [beta (*β*), 0.982; 95% confidence interval (CI), 0.691–1.274; VIF, 2.127; *p* < 0.001] and mFisher scores (*β*, 0.607; 95% CI, 0.271–0.943; VIF, 2.101; *p* < 0.001).

**Table 2:** Bivariate correlation assessments of admission serum PTEN-induced putative kinase 1 levels and 90-day Glasgow Outcome Scale scores after aneurysmal subarachnoid hemorrhage.

Parameters	Serum PINK1 levels		GOS scores	
	*ρ*	*p*-value	*ρ*	*p*-value
Gender (male/female)	0.060	0.397	−0.029	0.687
Age (years)	0.092	0.195	−0.230	0.001
Cigarette smoking	−0.056	0.427	−0.035	0.620
Alcohol consumption	−0.071	0.319	−0.045	0.528
Hyperlipidemia	−0.049	0.490	−0.058	0.412
Diabetes mellitus	0.074	0.298	−0.039	0.587
Hypertension	−0.010	0.891	0.016	0.819
WFNS scores	0.594	<0.001	−0.606	<0.001
Modified Fisher scores	0.559	<0.001	−0.596	<0.001
Aneurysmal position (posterior/anterior circulation)	0.114	0.108	−0.009	0.896
Aneurysmal shape (cystic/others)	0.076	0.284	0.077	0.279
Aneurysmal diameter (<10 mm/≥10 mm)	0.034	0.627	−0.003	0.970
Methods for securing aneurysms (clipping/endovascular intervention)	−0.061	0.391	0.055	0.493
Acute hydrocephalus	0.145	0.040	−0.164	0.020
Intraventricular extension of bleeding	0.213	0.002	−0.212	0.002
External ventricular drain	0.137	0.053	−0.188	0.008
Delayed cerebral ischemia	—	—	−0.481	<0.001
Admission time since stroke (h)	0.025	0.727	−0.050	0.484
Sampling time since stroke (h)	0.023	0.747	−0.044	0.534
Systolic arterial pressure (mmHg)	0.121	0.086	−0.051	0.475
Diastolic arterial pressure (mmHg)	0.135	0.055	−0.006	0.930
Blood glucose levels (mmol/L)	0.131	0.064	−0.188	0.008
Blood leucocyte counts (×10^9^/L)	−0.008	0.909	−0.004	0.960
Admission serum PINK1 levels (ng/mL)	—	—	−0.566	<0.001

Correlations were determined via the Spearman’s rank correlation test. PINK1, PTEN-induced putative kinase 1; WFNS, World Federation of Neurological Surgeons scale; GOS, Glasgow Outcome Scale.

### 3.3 Serum PINK1 levels and 90-day functional outcome

Ninety-day GOS scores were significantly related to serum PINK1 levels from admission to day 14 after aSAH (all *p* < 0.01; Supplementary Table 3). In addition, 90-day GOS scores of all patients were substantially pertinent to serum PINK1 levels at admission (*p* < 0.001; Supplementary Figure 7). Among all patients, admission serum PINK1 levels, age, WFNS scores, mFisher scores, acute hydrocephalus, intraventricular accumulation of bleeding, DCI, external ventricular drainage and blood glucose levels were highly correlated with 90-day GOS scores (all *p* < 0.05; Table 2). When the abovementioned nine factors were integrated into the multivariate linear regression model, WFNS scores (*β*, −0.245; 95% CI, −0.393 to −0.097; VIF, 2.520; *p* = 0.001), mFisher scores (β, −0.298; 95% CI, −0.461 to −0.134; VIF, 2.276; *p* < 0.001), admission serum PINK1 levels (*β*, −0.105; 95% CI, −0.161 to −0.049; VIF, 1.814; *p* < 0.001), and DCI (*β*, −0.325; 95% CI, −0.585 to −0.065; VIF, 1.369; *p* = 0.014) had independent correlations with 90-day GOS scores after aSAH.

Among five subgroups based on GOS, lower GOS scores subgroups had, higher serum PINK1 levels were since admission up to day 14 after aSAH (all *p* < 0.05; Supplementary Table 3). In addition, the identical condition occurred in all patients (*p* < 0.001; Supplementary Figure 8). Moreover, admission serum PINK1 levels, age, WFNS scores, mFisher scores, intraventricular expansion of bleeding, DCI and blood glucose levels were markedly different among the five subgroups from all patients (all *p* < 0.05; Table 3). When the aforementioned seven parameters were entered into the ordinal regression model, the WFNS scores (*β*, −0.541; 95% CI, −0.870 to −0.212; VIF, 2.518; *p* = 0.001), mFisher scores (*β*, −0.561; 95% CI, −0.921 to −0.202; VIF, 2.230; *p* = 0.002), admission serum PINK1 levels (*β*, −0.247; 95% CI, −0.372 to −0.123; VIF, 1.796; *p* = 0.001), and DCI (*β*, −0.711; 95% CI, −1.267 to −0.154; VIF, 1.331; *p* = 0.012) remained independently associated with ordinal GOS scores.

**Table 3:** Distinctions of baseline parameters across ordinal Glasgow Outcome Scale scores following aneurysmal subarachnoid hemorrhage.

Parameters	Ordinal GOS scores					
	1	2	3	4	5	*p*-value
Counts	16	24	37	75	49	
Gender (male/female)	7/9	12/12	17/20	34/41	21/28	0.986
Age (years)	54 (45–56)	56 (50–65)	51 (44–61)	52 (45–57)	45 (43–53)	0.006
Cigarette smoking	3 (18.8%)	9 (37.5%)	10 (27.0%)	24 (32.0%)	11 (22.4%)	0.545
Alcohol consumption	6 (37.5%)	8 (33.3%)	10 (27.0%)	24 (32.0%)	13 (26.5%)	0.892
Hyperlipidemia	4 (25.0%)	10 (41.7%)	10 (27.0%)	17 (22.7%)	13 (26.5%)	0.496
Diabetes mellitus	2 (12.5%)	2 (8.3%)	3 (8.1%)	3 (4.0%)	4 (8.2%)	0.731
Hypertension	3 (18.8%)	5 (20.8%)	5 (13.5%)	17 (22.7%)	9 (18.4%)	0.844
WFNS scores	4 (4–5)	4 (3–4)	3 (3–4)	3 (2–3)	2 (1–2)	<0.001
Modified Fisher scores	4 (3–4)	3 (2–3)	3 (2–3)	2 (1–3)	1 (1–2)	<0.001
Aneurysmal position (posterior/anterior circulation)	5/11	6/18	11/26	19/56	15/34	0.954
Aneurysmal shape (cystic/others)	10/6	20/4	28/9	66/9	39/10	0.147
Aneurysmal diameter (<10 mm/≥10 mm)	7/9	16/8	20/17	49/26	26/23	0.350
Methods for securing aneurysms (clipping/endovascular intervention)	4/12	12/12	16/21	28/47	24/25	0.379
Acute hydrocephalus	4 (25.0%)	6 (25.0%)	8 (21.6%)	11 (14.7%)	4 (8.2%)	0.242
Intraventricular extension of bleeding	5 (31.3%)	6 (25.0%)	10 (27.0%)	16 (21.3%)	2 (4.1%)	0.029
External ventricular drain	4 (25.0%)	5 (20.8%)	6 (16.2%)	9 (12.0%)	2 (4.1%)	0.125
Delayed cerebral ischemia	12 (75.0%)	14 (58.3%)	15 (40.5%)	13 (17.3%)	2 (4.1%)	<0.001
Admission time since stroke (h)	6.9 (3.2–14.6)	7.4 (4.2–15.3)	9.6 (7.0–15.0)	8.2 (3.4–12.9)	6.6 (4.1–10.3)	0.416
Sampling time since stroke (h)	7.8 (3.9–15.2)	8.0 (5.0–16.1)	10.1 (7.9–15.7)	9.3 (4.1–13.4)	8.0 (5.1–11.1)	0.382
Systolic arterial pressure (mmHg)	152 (122–177)	142 (120–154)	131 (119–154)	135 (125–147)	136 (125–151)	0.687
Diastolic arterial pressure (mmHg)	91 (77–104)	88 (79–93)	86 (75–95)	89 (81–94)	88 (77–92)	0.686
Blood glucose levels (mmol/L)	16.7 (11.1–19.6)	13.1 (9.3–17.3)	10.6 (8.5–15.3)	11.5 (8.6–14.5)	10.7 (7.8–13.8)	0.045
Blood leucocyte counts (×10^9^/L)	6.3 (4.6–10.2)	8.7 (5.6–11.0)	8.0 (5.9–11.3)	7.2 (5.3–10.4)	7.7 (5.2–9.3)	0.848
Admission serum PINK1 levels (ng/mL)	7.8 (7.4–8.6)	8.4 (6.5–8.8)	6.8 (4.2–8.0)	4.9 (3.8–6.7)	3.8 (2.4–4.0)	<0.001

Data were reported as medians with 25th–75th percentiles or counts (proportions) as appropriate. The chi-square test or Kruskal–Wallis *H* test, as applicable, was applied for statistical analyses. PINK1, PTEN-induced putative kinase 1; WFNS, World Federation of Neurological Surgeons scale; GOS, Glasgow Outcome Scale.

Patients with poor prognosis, in contrast to those with good prognosis, possessed notably raised serum PINK1 levels since admission up to day 14 subsequent to aSAH (all *p* < 0.01; Supplementary Table 4); and serum PINK1 levels at admission had AUC similar to those from day 1 to 14 post aSAH (all *p* > 0.05; Supplementary Table 4). As shown in Supplementary Figure 9, serum PINK1 levels at admission were significantly higher in patients with poor prognosis than in those with good prognosis among all patients (*p* < 0.001). Moreover, poor prognosis was effectively predicted by admission serum PINK1 levels, and the level above 6.6 ng/mL prognosticated poor prognosis with the maximum Youden index (Supplementary Figure 10). Admission serum PINK1 levels were linearly linked to the probability of poor prognosis (nonlinear *p* > 0.05; Supplementary Figure 11).

As presented in Table 4, patients with poor prognosis, relative to those with good prognosis, tended to be significantly older; were apt to have substantially higher WFNS and mFisher scores; were prone to have markedly higher percentages of acute hydrocephalus, DCI, intraventricular extension of bleeding, and external ventricular drainage; and were likely to have notably higher blood glucose levels and admission serum PINK1 levels (all *p* < 0.05). By forcing the preceding nine variables into the binary logistic regression model, WFNS scores [odds ratio (OR), 1.515; 95% CI, 1.137–2.323; VIF, 1.782; *p* = 0.001], mFisher scores (OR, 1.652; 95% CI, 1.040–2.623; VIF, 2.117; *p* = 0.003), admission serum PINK1 levels (OR, 1.249; 95% CI, 1.071–1.456; VIF, 1.761; *p* = 0.005), and DCI (OR, 1.822; 95% CI, 1.037–3.622; VIF, 1.441; *p* = 0.017) emerged as the four independent predictors of poor prognosis at 90-days after aSAH (*p* = 0.331; via Hosmer and Lemeshow test).

**Table 4:** Baseline characteristics pertinent to poor prognosis and delayed cerebral ischemia after aneurysmal subarachnoid hemorrhage.

Parameters	Poor prognosis			Delayed cerebral ischemia		
	Yes	No	*p*-value	Presence	Absence	*p*-value
Counts	77	124		56	145	
Gender (male/female)	36/41	55/69	0.740	24/32	67/78	0.669
Age (years)	53.6 ± 10.0	50.6 ± 9.7	0.037	52.6 ± 9.5	51.4 ± 10.1	0.430
Cigarette smoking	22 (28.6%)	35 (28.2%)	0.958	13 (23.2%)	44 (30.3%)	0.315
Alcohol consumption	24 (31.2%)	37 (29.8%)	0.842	15 (26.8%)	46 (31.7%)	0.495
Hyperlipidemia	24 (31.2%)	30 (24.2%)	0.278	16 (28.6%)	38 (26.2%)	0.735
Diabetes mellitus	7 (9.1%)	7 (5.6%)	0.351	3 (5.4%)	11 (7.6%)	0.578
Hypertension	13 (16.9%)	26 (21.0%)	0.477	8 (14.3%)	31 (21.4%)	0.254
WFNS scores	3 (3–4)	2 (2–3)	<0.001	4 (3–4)	2 (2–3)	<0.001
Modified Fisher scores	3 (2–3)	2 (1–2)	<0.001	3 (2–3)	2 (1–3)	<0.001
Aneurysmal position (posterior/anterior circulation)	22/55	34/90	0.859	17/39	39/106	0.624
Aneurysmal shape (cystic/others)	58/19	105/19	0.100	48/8	115/30	0.299
Aneurysmal diameter (<10 mm/≥10 mm)	43/34	75/49	0.516	30/26	88/57	0.358
Methods for securing aneurysms (clipping/endovascular intervention)	32/45	52/72	0.958	25/31	59/86	0.610
Acute hydrocephalus	18 (23.4%)	15 (12.1%)	0.036	15 (26.8%)	18 (12.4%)	0.014
Intraventricular extension of bleeding	21 (27.3%)	18 (14.5%)	0.026	18 (32.1%)	21 (14.5%)	0.005
External ventricular drain	15 (19.5%)	11 (8.9%)	0.029	14 (25.0%)	12 (8.3%)	0.002
Delayed cerebral ischemia	41 (53.2%)	15 (12.1%)	<0.001	—	—	—
Admission time since stroke (h)	8.4 (4.2–15.2)	8.0 (3.9–12.2)	0.259	9.2 (5.7–13.5)	8.0 (3.8–12.8)	0.224
Sampling time since stroke (h)	9.4 (5.1–16.1)	8.7 (4.6–12.9)	0.269	9.9 (6.5–13.9)	8.8 (4.6–13.3)	0.232
Systolic arterial pressure (mmHg)	140.3 ± 27.9	137.4 ± 20.6	0.390	140.7 ± 25.0	137.6 ± 23.1	0.409
Diastolic arterial pressure (mmHg)	86.7 ± 12.5	87.5 ± 10.8	0.638	87.8 ± 11.4	86.9 ± 11.5	0.631
Blood glucose levels (mmol/L)	12.2 (8.6–17.5)	11.1 (7.9–14.4)	0.043	13.7 (8.7–17.7)	11.1 (8.1–14.3)	0.022
Blood leucocyte counts (×10^9^/L)	8.0 (5.5–11.2)	7.5 (5.3–10.4)	0.882	8.0 (6.1–11.3)	7.5 (5.1–10.8)	0.394
Admission serum PINK1 levels (ng/mL)	7.8 (5.0–8.5)	4.0 (3.1–5.8)	<0.001	7.7 (5.5–8.7)	4.1 (3.1–6.5)	<0.001

Medians with 25th–75th percentiles, means (standard deviations) or counts (proportions), as applicable, were reported for showing data; and the *χ*^2^ test, Fisher’s exact test, *t*-test or Mann–Whitney *U*-test, as suitable, was operated for statistical assessments. PINK1, PTEN-induced putative kinase 1; WFNS, World Federation of Neurological Surgeons scale.

**Figure 2:**
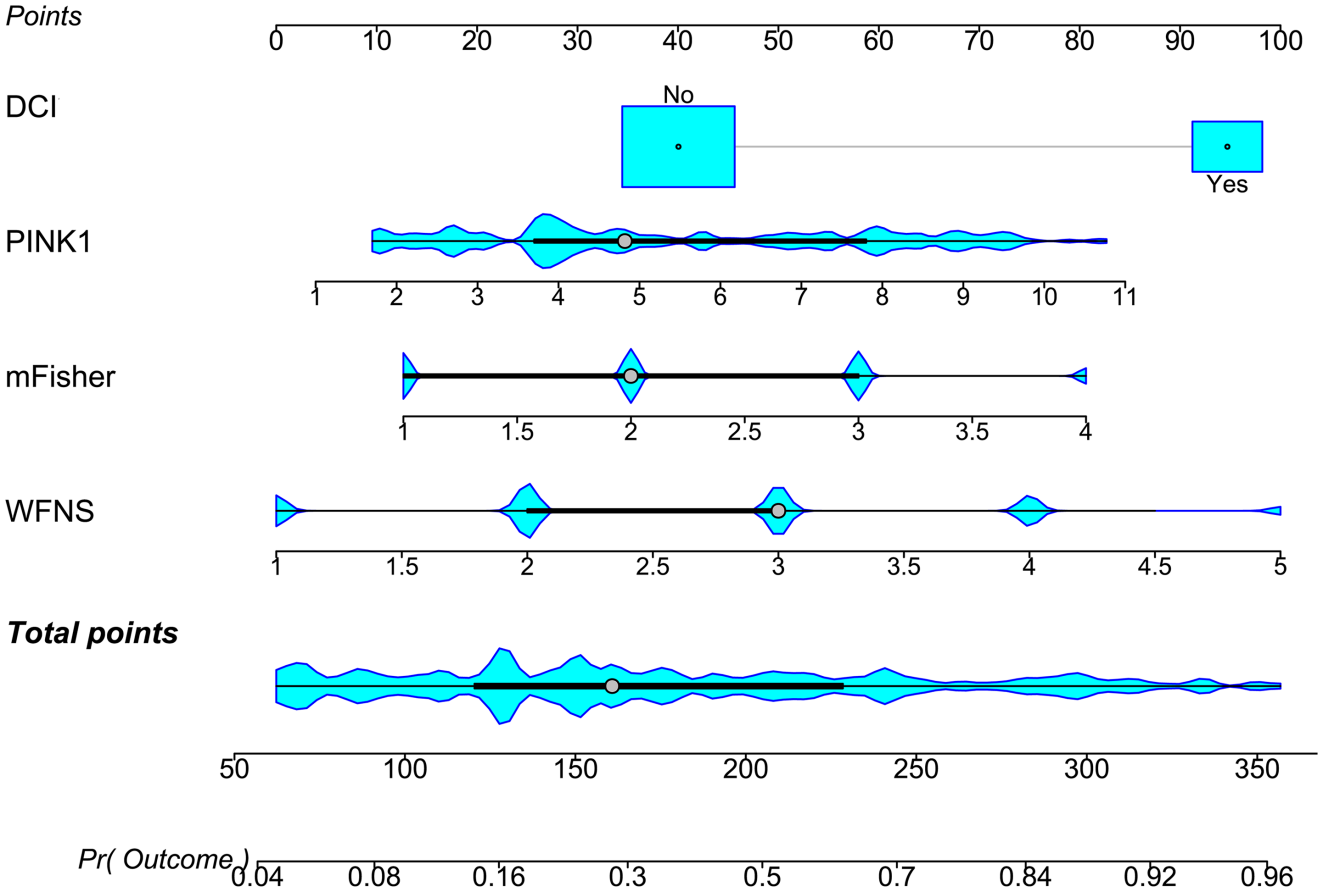
Nomogram of prognosis model following aneurysmal subarachnoid hemorrhage. The modified Fisher scale, World Federation of Neurosurgical Societies scale, serum PTEN-induced putative kinase 1, and delayed cerebral ischemia were merged to establish a model for predicting a poor prognosis. The respective scores were summed to estimate the possibility of poor prognosis in patients with aneurysmal subarachnoid hemorrhage. PINK1, PTEN-induced putative kinase 1; WFNS, World Federation of Neurosurgical Societies scale; mFisher, modified Fisher; DCI, delayed cerebral ischemia.

**Figure 3:**
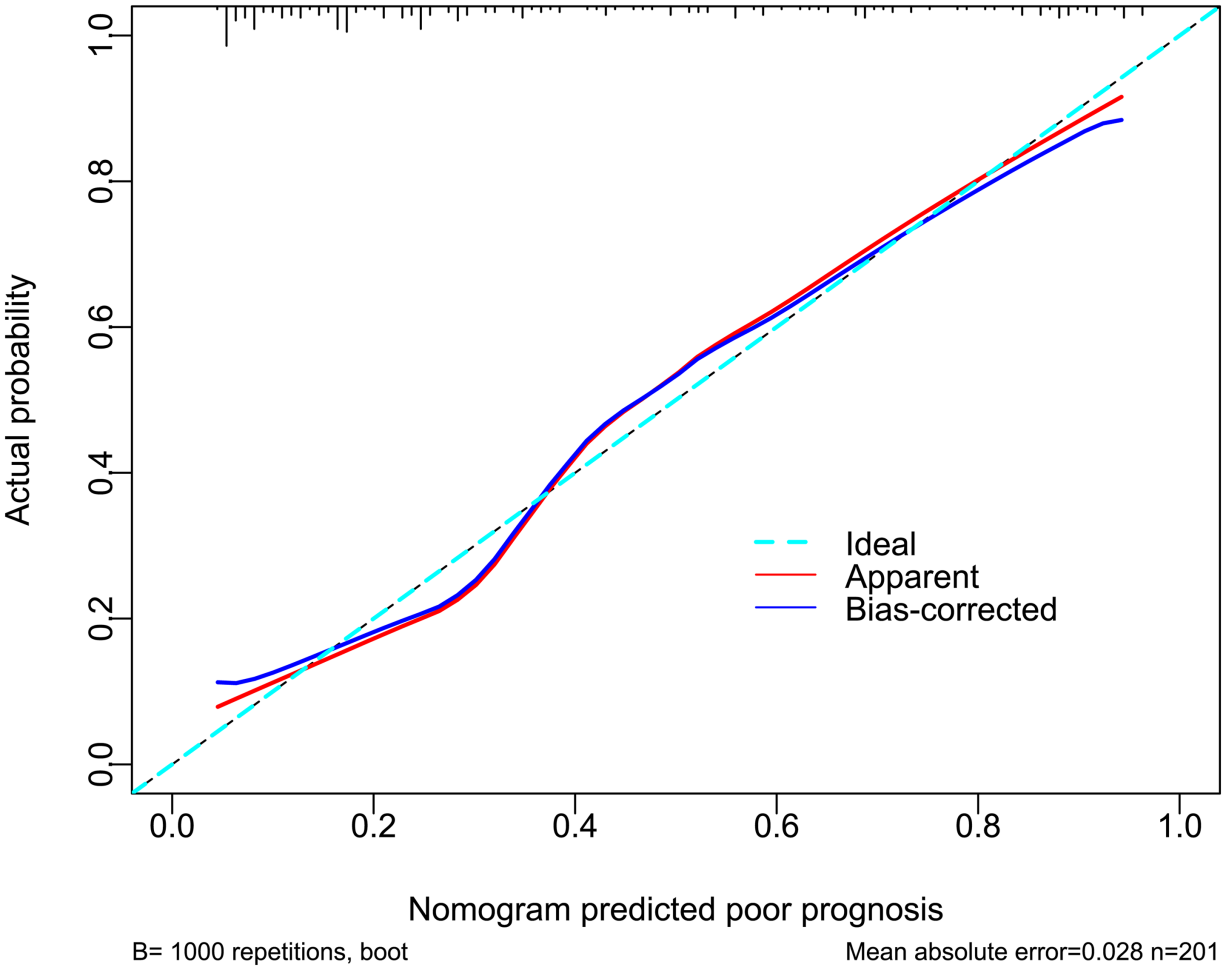
Stability of prognosis model in aneurysmal subarachnoid hemorrhage. The modified Fisher scale, World Federation of Neurosurgical Societies scale, serum PTEN-induced putative kinase 1, and delayed cerebral ischemia were combined to construct a model for predicting poor prognosis. The model showed a good stability in prognosis anticipation of patients with aneurysmal subarachnoid hemorrhage by using the calibration curve analysis.

**Figure 4:**
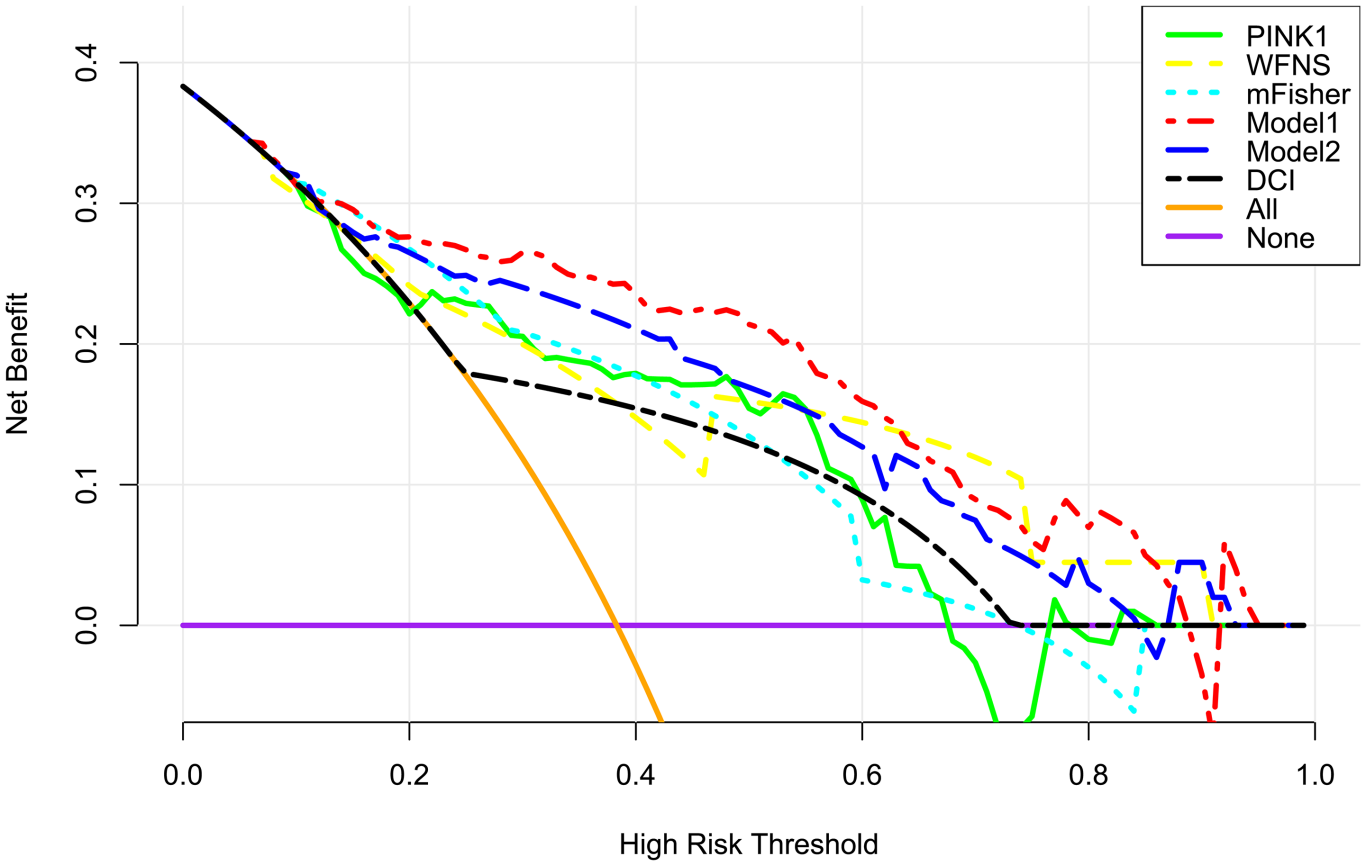
Clinical benefit of prognosis model in aneurysmal subarachnoid hemorrhage. The modified Fisher scale, World Federation of Neurosurgical Societies scale, serum PTEN-induced putative kinase 1, and delayed cerebral ischemia were integrated to build model 1 for predicting poor prognosis; the modified Fisher, World Federation of Neurosurgical Societies scale, and delayed cerebral ischemia were incorporated to configure the model 2. Among these six variables, the mode 1 displayed good clinical validity in the context of decision curve analysis. PINK1, PTEN-induced putative kinase 1; WFNS, World Federation of Neurosurgical Societies scale; mFisher, modified Fisher; DCI, delayed cerebral ischemia.

**Figure 5:**
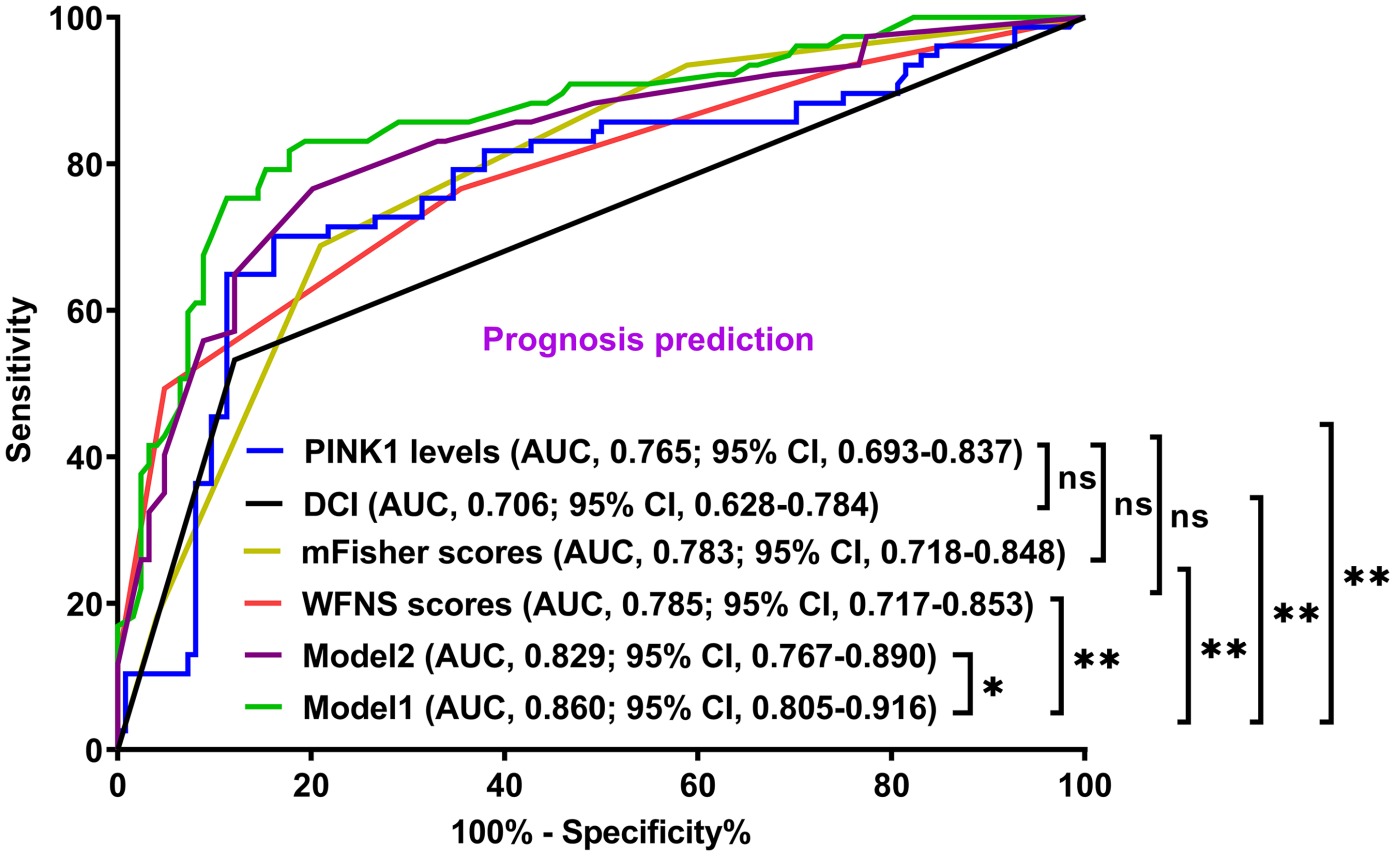
Prediction capability of prognosis model in aneurysmal subarachnoid hemorrhage. The modified Fisher scale, World Federation of Neurosurgical Societies scale, serum PTEN-induced putative kinase 1, and delayed cerebral ischemia were combined to form model 1; the modified Fisher scale, World Federation of Neurosurgical Societies scale, and delayed cerebral ischemia were consolidated to construct model 2. Among these six variables, model 1 had a satisfactory discrimination efficiency based on the receiver operating characteristic curve. PINK1, PTEN-induced putative kinase 1; WFNS, World Federation of Neurosurgical Societies scale; mFisher, modified Fisher; DCI, delayed cerebral ischemia; AUC, area under the curve; 95% CI, 95% confidence interval; ns, non-significant. ^**^p < 0.01, ^*^p < 0.05.

A prognostic model was created by merging the preceding four independent predictors. The model was delineated in form of the nomogram in which the cumulative scores were computed for estimating poor prognosis risk (Figure 2). A satisfactory model fit was confirmed by calibration curve analysis (Figure 3), and its clinical effectiveness was proven with the assistance of the decision curve assessment (Figure 4). Within the framework of the ROC curve approach, admission serum PINK1 levels exhibited AUC similar to WFNS scores, mFisher scores, and DCI (all *p* > 0.05; Figure 5), and the model had good discrimination efficiency with the obviously higher AUC than admission serum PINK1 levels, WFNS scores, mFisher scores, and DCI alone, as well as the combination of the latter three predictors (all *p* < 0.05; Figure 5).

### 3.4 Serum PINK1 levels and DCI, and the mediation role of DCI on prognostic association

Patients with the development of DCI, as opposed to those without, had markedly heightened serum PINK1 levels at serial time points post aSAH (all *p* < 0.05; Supplementary Table 5); admission serum PINK1 levels showed AUC similar to those from day 1 to day 10 (all *p* > 0.05; Supplementary Table 5), as well as had markedly higher AUC than that on day 14 post aSAH (*p* < 0.05; Supplementary Table 5). Also, admission serum PINK1 levels were substantially higher in patients experiencing DCI than in the other remainders (*p* < 0.001, Supplementary Figure 12). Moreover, admission serum PINK1 levels efficaciously identified the DCI likelihood, and the level >6.2 ng/mL predicted DCI with the maximum Youden index at 0.470 (Supplementary Figure 13). A linear relationship was observed between serum PINK1 levels at admission and DCI risk (nonlinear *p* > 0.05; Supplementary Figure 14).

As presented in Table 4, patients with DCI, in comparison to those without, had significantly higher WFNS and mFisher scores, had substantially higher proportions of acute hydrocephalus, intraventricular extension of bleeding and external ventricular drainage, and held significantly higher blood glucose and admission serum PINK1 levels (all *p* < 0.05). By adding all seven significant factors into the binary logistic regression model, the independent predictors of DCI were the WFNS score (OR, 1.572; 95% CI, 1.012–2.422; VIF, 1.980; *p* = 0.012), mFisher scores (OR, 1.588; 95% CI, 1.092–2.584; VIF, 2.024; *p* = 0.023), and admission serum PINK1 levels (OR, 1.193; 95% CI, 1.015–1.402; VIF, 1.501; *p* = 0.025), and the model had a good fit according to the Hosmer and Lemeshow test (*p* = 0.136).

**Figure 6:**
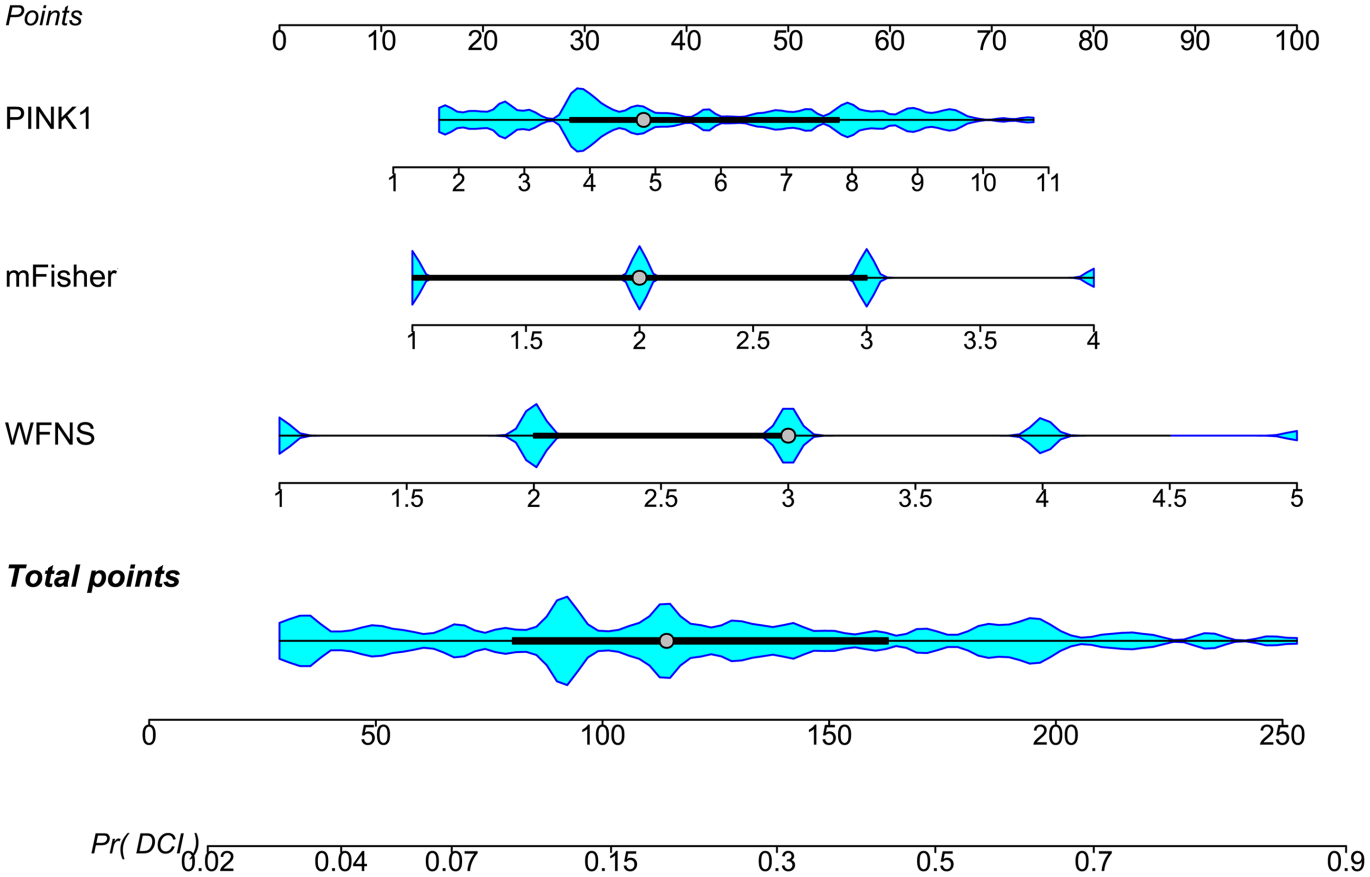
Nomogram describing combined model of delayed cerebral ischemia subsequent to aneurysmal subarachnoid hemorrhage. Three independent predictors of delayed cerebral ischemia, that is the modified Fisher scale, World Federation of Neurosurgical Societies scale, and serum PTEN-induced putative kinase 1, were consolidated to create a prediction model. Aggregate scores calculated from all variables were adopted to analyze the risk of delayed cerebral ischemia in patients with aneurysmal subarachnoid hemorrhage. PINK1, PTEN-induced putative kinase 1; WFNS, World Federation of Neurosurgical Societies scale; mFisher, modified Fisher; DCI, delayed cerebral ischemia.

**Figure 7:**
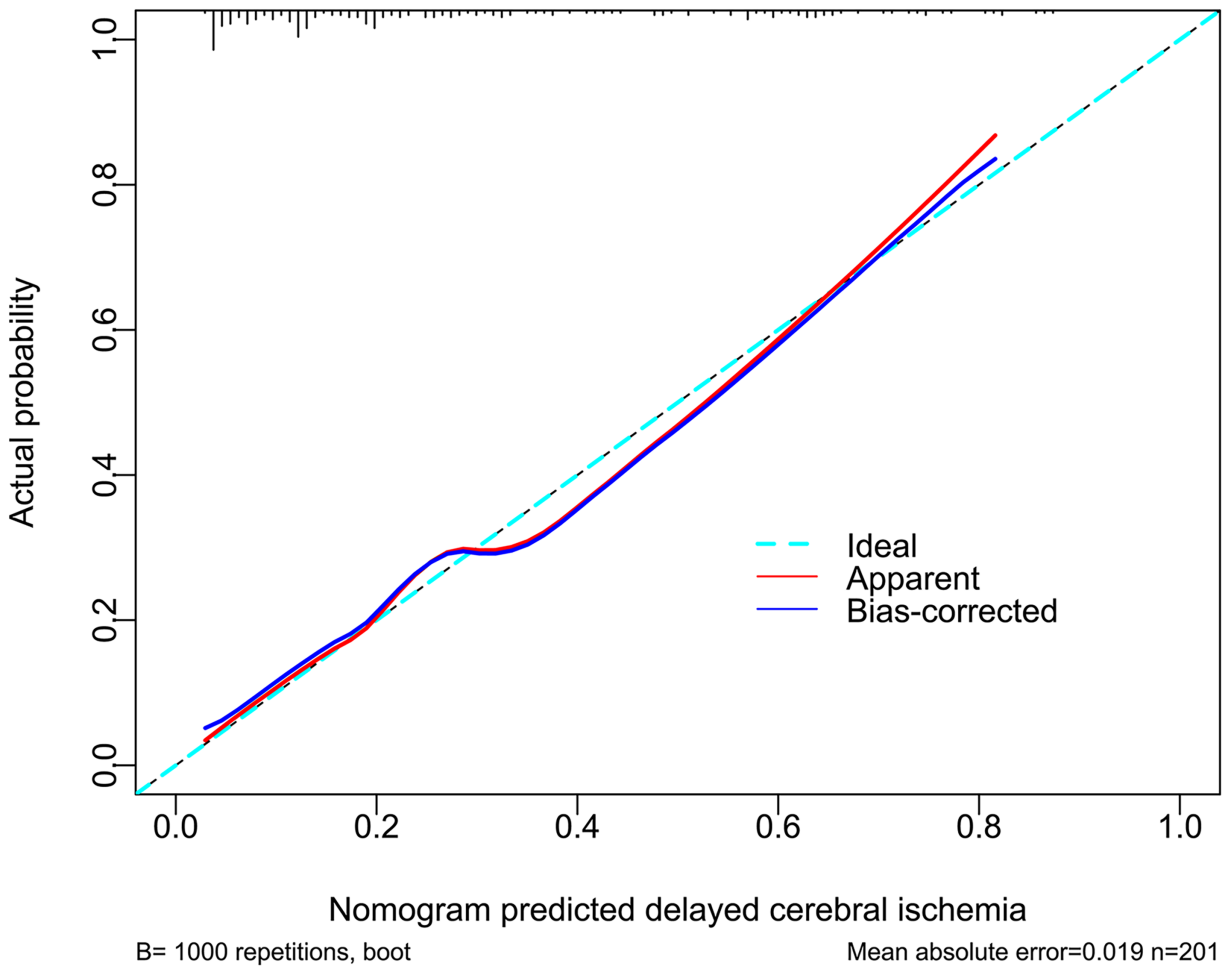
Affirmation of model stability in forecasting delayed cerebral ischemia following aneurysmal subarachnoid hemorrhage. The modified Fisher scale, World Federation of Neurosurgical Societies scale, and serum PTEN-induced putative kinase 1 were put together to configure the model for foretelling delayed cerebral ischemia. The model maintained a good stability in predicting delayed cerebral ischemia in patients with aneurysmal subarachnoid hemorrhage via calibration curve analysis.

**Figure 8:**
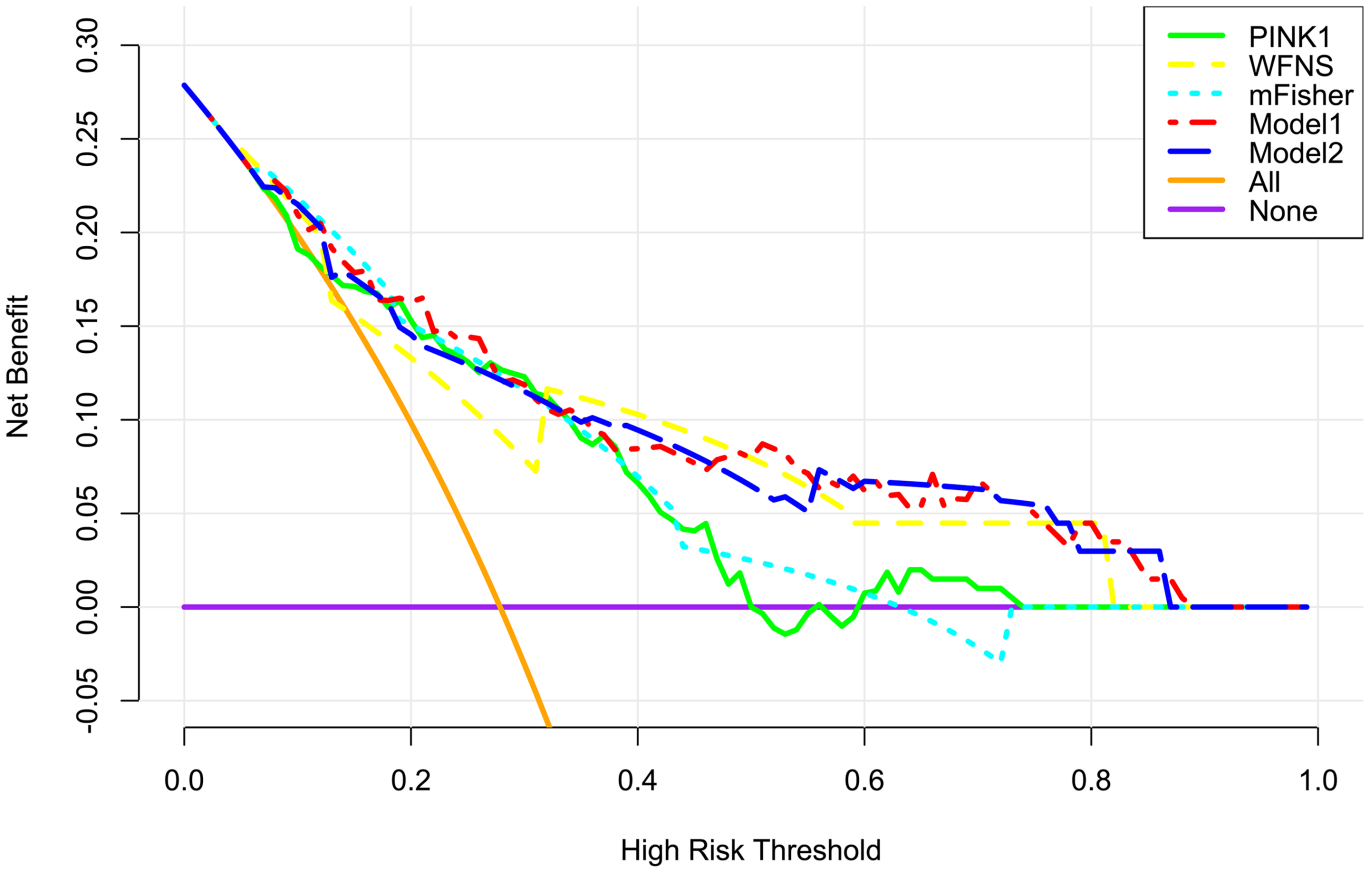
Verification of clinical effectiveness of the merged model in predicting delayed cerebral ischemia secondary to aneurysmal subarachnoid hemorrhage. Model 1 comprised the modified Fisher scale, World Federation of Neurosurgical Societies scale, and serum PTEN-induced putative kinase 1; Model 2 was composed of the modified Fisher scale and World Federation of Neurosurgical Societies scale. Among these five variables, the mode1 was comparatively beneficial in forecasting delayed cerebral ischemia in patients by employing decision curve analysis. PINK1, PTEN-induced putative kinase 1; WFNS, World Federation of Neurosurgical Societies scale; mFisher, modified Fisher.

**Figure 9:**
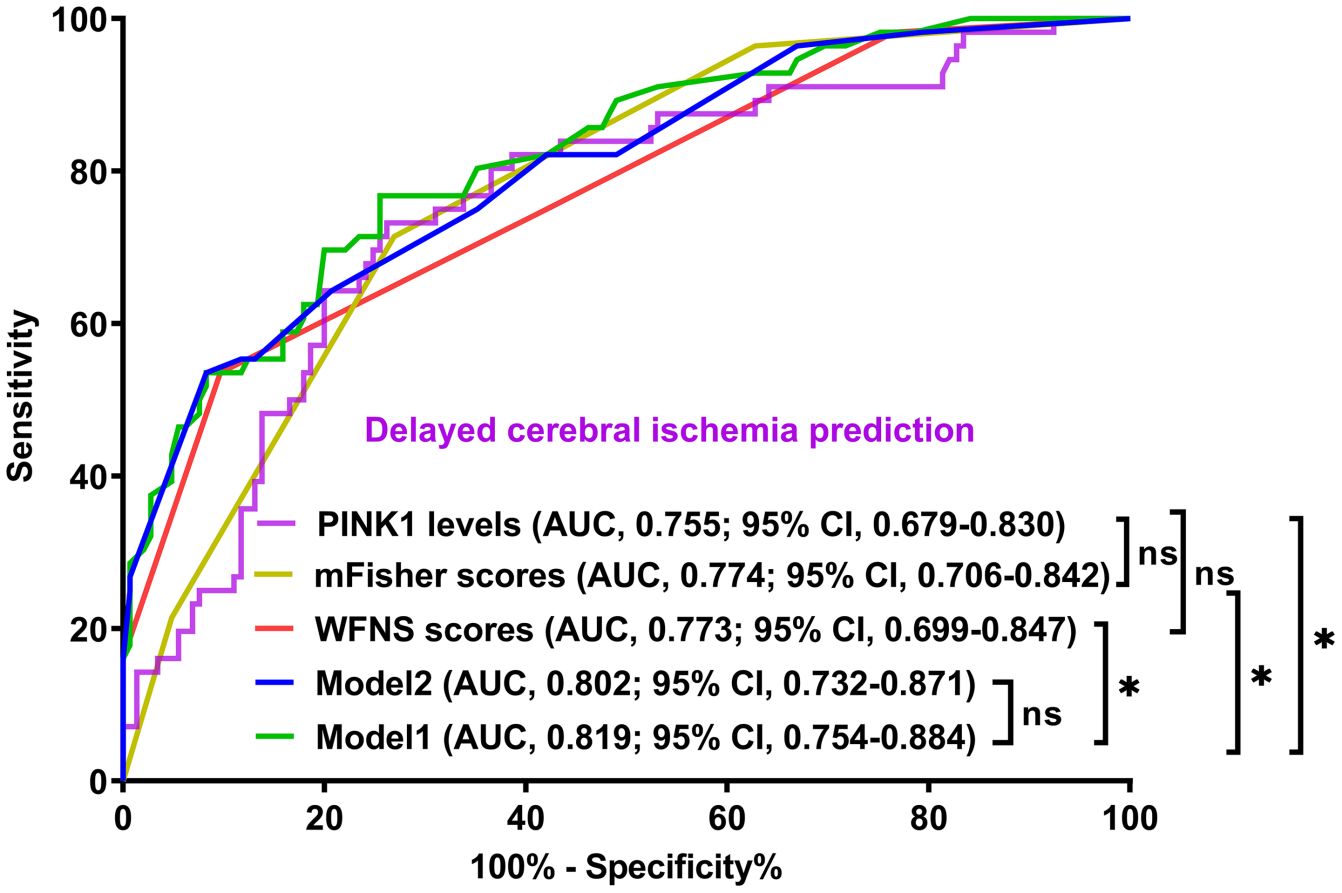
Demonstration of model efficiency in distinguishing risk of delayed cerebral ischemia secondary to aneurysmal subarachnoid hemorrhage. The modified Fisher scale, World Federation of Neurosurgical Societies scale, and serum PTEN-induced putative kinase 1 composed model 1; the modified Fisher scale and World Federation of Neurosurgical Societies Scale constituted model 2. The predictive ability of serum PTEN-induced putative kinase 1 levels resembled those of the modified Fisher scale and World Federation of Neurosurgical Societies scales. The mode1 had discrimination efficiency surpassing those of the modified Fisher scale, World Federation of Neurosurgical Societies scale, and serum PTEN-induced putative kinase 1, but not transcending that of model 2 according to the receiver operating characteristic curve. PINK1, PTEN-induced putative kinase 1; WFNS, World Federation of Neurosurgical Societies scale; mFisher, modified Fisher; AUC, area under the curve; 95% CI, 95% confidence interval; ns, non-significant. ^*^p < 0.05.

The three predictors of DCI, that is WFNS scores, mFisher scores and admission serum PINK levels, were incorporated to build combined model to discriminate the probability of DCI. The model was visualized via the nomogram for dynamically monitoring the probability of DCI (Figure 6). The model was comparatively stable within the framework of the calibration curve analysis (Figure 7) and was relatively clinically valid in the context of decision curve analysis (Figure 8). Under the ROC curve, admission serum PINK1 levels had AUC comparable to WFNS and mFisher scores (both *p* > 0.05; Figure 9), and the model owned markedly higher AUC than admission serum PINK1 levels, WFNS scores, and mFisher scores alone (all *p* < 0.05; Figure 9), but not significantly higher AUC than the combination of the latter two predictors (*p* > 0.05; Figure 9).

**Figure 10:**
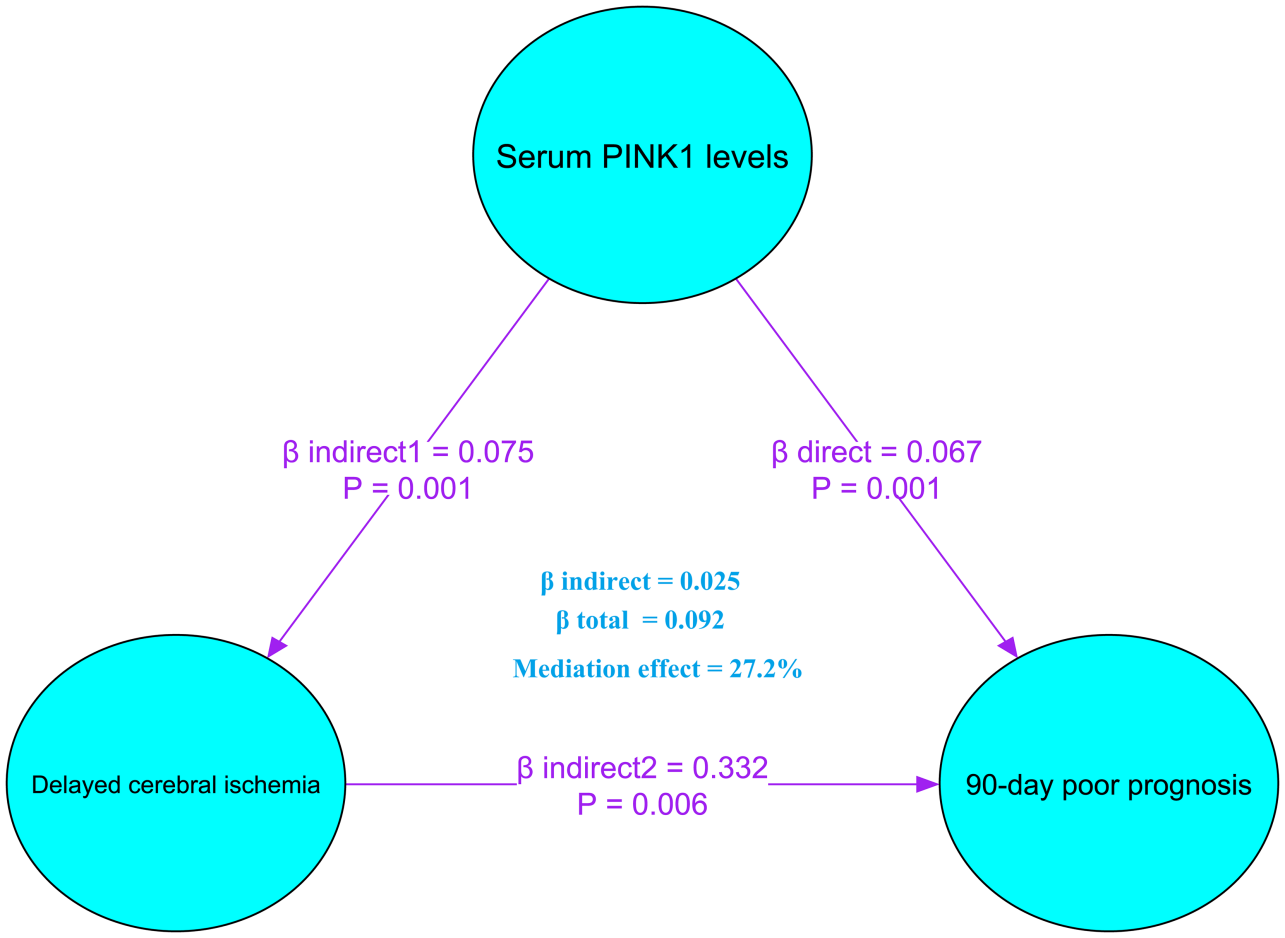
Role of delayed cerebral ischemia in mediating the association of serum PTEN-induced putative kinase 1 levels with poor prognosis after aneurysmal subarachnoid hemorrhage. Mediation analysis showed that delayed cerebral ischemia mediated 27.2% of the associations between serum PTEN-induced putative kinase 1 levels and poor prognosis after aneurysmal subarachnoid hemorrhage. PINK1, PTEN-induced putative kinase 1.

**Figure 11:**
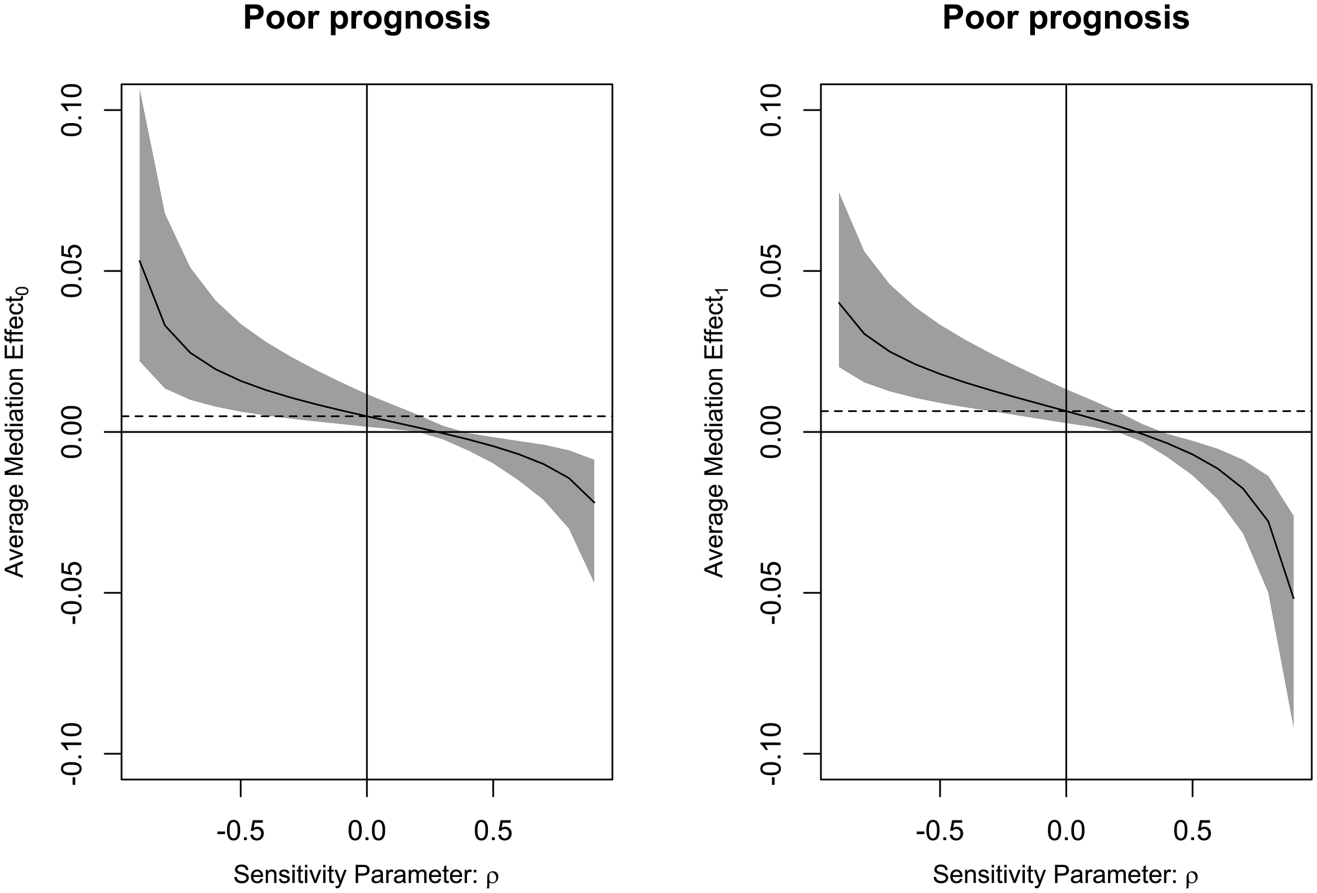
Analysis of the strength of the mediation effect of delayed cerebral ischemia. By employing sensitivity analysis, it was revealed that mediating effect of delayed cerebral ischemia was relatively robust on the association of serum PTEN-induced putative kinase 1 levels with poor prognosis following aneurysmal subarachnoid hemorrhage under both control and treatment conditions (both *ρ* = 0.300).

Mediation analysis showed that DCI partially mediated the association between serum PINK1 level at admission and poor prognosis, with the mediation effect of 27.2% (Figure 10). Moreover, this sort of mediation effect was robust when adopting sensitivity analysis under control and treatment conditions (both *ρ* = 0.300; Figure 11).

## 4 Discussion

To the best of our knowledge, serum PINK1 levels have not yet been investigated in humans following aSAH. Here, we found that serum-based PINK1 levels were virtually increased after aSAH, peaking on day 3. In addition, serum levels of this protein were independently correlated with sickness severity. Also, serum levels were independently associated with DCI and poor prognosis at 90 days after aSAH. Moreover, serum PINK1 levels had high predictive ability for DCI and poor prognosis, and models encompassing serum PINK1 showed good clinical values. Notably, the association between serum PINK1 levels and poor prognosis was mediated, in part, by DCI. Overall, these findings suggest that serum PINK1 could be a potential prognostic biomarker of aSAH.

To depict temporal alterations of serum PINK1 levels post-aSAH, a cohort of patients volunteered to obtain blood samples at seven time points. The baseline parameters of this group of patients were analogous to those of all patients, signifying that this portion of patients could statistically represent the whole group of patients in the current study. Here, serum PINK1 levels were elevated after aSAH, with the highest value on day 3, and were still significantly higher even up to day 14 after aSAH than those of controls. Moreover, the PINK1 levels were similar between all patients and this part of patients. Reportedly, PINK1 mRNA expressions were markedly upregulated in the cerebral tissues of patients with intracerebral hemorrhage (22). In another study, PINK1 levels were notably elevated in the cerebrospinal fluid and serum samples of patients with multiple sclerosis or Alzheimer’s disease (23, 24). Collectively, these results suggest that blood PINK1 levels may be significantly elevated following aSAH.

PINK1 is abundant in the mitochondria and functionally sustains mitochondrial integrity by selectively cleaning up intercellular debris via autophagy (29). Through animal experiments of intracerebral hemorrhage, ischemic stroke, SAH and traumatic brain injury, PINK1 is well acknowledged as a protective factor against extensive forms of acute and chronic brain injuries (18–21). Moreover, PINK1 expressions were profoundly upregulated in the cerebral cortices of rats subjected to SAH (21). Also, there was a substantial elevation of brain PINK1 mRNA expressions in patients with intracerebral hemorrhage (22). Thus, PINK1 levels may be elevated in response to acute brain injury. Moreover, blood PINK1 may at least partially stem from the central nervous system, given that PINK1 can exist in the cerebrospinal fluid of humans with multiple sclerosis or Alzheimer’s disease (23, 24). Nevertheless, autophagy is indispensable for protecting cellular functions (30), and aSAH is regularly accompanied by systemic injury (31, 32). Thus, it is not excluded that blood PINK1 may be fractionally derived from peripheral cells.

In the current study, the peak of serum PINK1 levels occurred on the third day after aSAH, and then the levels dropped until the fourteenth day, but were still higher than those of controls. By comparing the time course characteristics of other brain injury markers, e.g., S100B, neuron-specific enolase, glial fibrillary acidic protein, ubiquitin carboxy-terminal hydrolase L1 and neurofilament light, it is revealed that the preceding biomarkers’ levels in peripheral blood generally return to normal status within 14 days following acute brain injury (33, 34). Hence, temporal dynamic features of serum PINK1 levels following aSAH may be peculiar and the continuous increase of serum PINK1 levels until at least 14 days may reflect chronic injury. It is meaningful to in future measure serum PINK1 levels at days 21 and even 28 post-aSAH, so as to clearly define the time window for “serum PINK1 levels’ returning to normal value.”

As for the relationship between PINK1 levels and disease severity, cerebral PINK1 mRNA expression levels were tightly correlated with clinical scores and hematoma size following acute intracerebral hemorrhage (22), and PINK1 levels in cerebrospinal fluid and serum were highly relevant to the memory, executive function, and language domains of patients with Alzheimer’s disease (23). WFNS and mFisher scores are two paramount severity metrics of aSAH (8, 9). In the current group of 87 patients with aSAH, serum PINK1 levels at the seven time points were obviously in positive proportion to the initial WFNS and mFisher scores. In all 201 patients, serum PINK1 levels at admission were independently associated with the initial WFNS and mFisher scores. In summary, our study provides statistical evidence to indicate that serum PINK1 may be a useful biomarker for facilitating severity assessment in the clinical practice of aSAH management.

Clinically, the GOS has been extensively utilized in the assessment of neurological function in the daily lives of patients with acute brain injury, including aSAH (10). The post-aSAH 90-day GOS scores of 87 patients were strongly associated with serum PINK1 levels at serial time points, and prognostic value of admission serum PINK1 levels was analogous to those at the other time points, indicating that serum PINK1 levels at admission could play an appealing role as a prognostic biomarker of aSAH. In the next step, serum PINK1 levels at admission were independently associated with continuous, ordinary, and binary GOS scores in all 201 patients. Risk of poor prognosis were effectively discriminated by admission serum PINK1 levels. The model encompassing the four independent predictors (i.e., DCI, WFNS, mFisher, and serum PINK1) showed good performance in predicting poor prognosis of aSAH according to calibration curve, decision curve and ROC curve analyses. Therefore, these auxiliary data have proffered further supportive evidence to affirm the satisfactory prognostic ability of serum PINK1 in aSAH.

Although the combined model integrating WFNS, mFisher, DCI and serum PINK1 was in possession of AUC at 0.860, which significantly surpassed those of WFNS, mFisher and DCI alone (AUC = 0.785–0.829), but the incremental value of serum PINK1 on prognosis ability may be a little small in terms of AUC (the minimum value of subtracted AUC at 0.031). Such condition leads to the notion that serum PINK1 may not sufficient to clinically heighten prognosis capability in this entity of aSAH. Nevertheless, this finding has from the other perspective strengthen serum PINK1 as a potential prognostic biomarker of human aSAH. As such, patients can be stratified for different managements according to the cutoff value of serum PINK1 levels. Serum PINK1 levels above the threshold value means that those patients may be at risk of poor prognosis and therefore should be actively monitored and even aggressively and correspondingly treated. A single serum PINK1 test, relative to complex combined models, is marked by an easy measurement in consideration of convenient obtainability of blood samples. Therefore, serum PINK1 can hold the potential application during clinical work of aSAH in resource-limited regions.

DCI is a pivotal predictor of poor prognosis after aSAH (7). DCI is independently associated with poor prognosis in patients with aSAH. Autophagy is a vital process during DCI formation, although complex mechanisms remain to be elucidated (25, 26). In a previous study of aSAH patients, DCI cases versus non-DCI cases showed significantly increased PINK1 mRNA expression levels in cerebrospinal fluid samples (25, 26). Serum PINK1 levels at any pre-specified time point were significantly higher in patients with DCI than in those without DCI in the current study. Moreover, the serum PINK1 levels at admission were sufficient to differentiate DCI development from the others. Taken together, these data indicate that autophagy is linked to the appearance of DCI post-aSAH. Although admission serum PINK1 levels were independently associated with emergence of DCI, the predictive capability of the combined model was similar to that of WFNS combined with mFisher. The results mean that, in contrast to prognosis prediction, serum PINK1 may not hold a strong advantage for DCI prediction. Notably, there was a meaningful role of DCI in partially mediating the association between serum PINK1 levels and poor prognosis in this cohort of patients with aSAH. Hence, DCI may partially interpret the links between serum PINK1 levels and poor prognosis of aSAH. Although the detailed mechanisms of this phenomenon have not been addressed up to date, autophagy should be highlighted as a critical bridge of this type of link.

This study has several strengths and weaknesses. The strengths are in the following. First, to the best of our knowledge, circulating PINK1 levels after aSAH are measured for the first time, and it is found that serum PINK1 may represent a promising biochemical marker of potential clinical outcomes. Second, several multivariate analyses were implemented to prove the relevance of severity and outcomes to serum PINK1 levels so as to ensure that serum PINK1 may be a good prognostic biomarker of aSAH. The weaknesses are shown below. First, although seven time points were pre-established for sampling in this study, when serum PINK1 levels recover to normal status is not ascertained, so this should be investigated in the future. Second, sufficient statistical power has been offered to demonstrate the prognostic significance of serum PINK1 in aSAH, but any conclusion should be validated in a larger cohort study for good generalization.

## 5 Conclusion

Serum PINK1 levels remain significantly elevated up to at least 14 days after aSAH and are independently related to illness intensity, DCI and poor 90-day prognosis following aSAH as well. Poor prognosis and DCI can be well predicted using the models that integrate serum PINK1 levels. DCI may partially explain the association between serum PINK1 levels and poor prognosis after aSAH. Taken together, serum PINK1 could be a prognostic biomarker to enable risk stratification of aSAH.

## Data Availability

The raw data supporting the conclusions of this article will be made available by the authors, without undue reservation.
