# Supplementary material for: Longitudinal change and prognostic significance of serum PINK1 levels, and mediation role of delayed cerebral ischemia in human aneurysmal subarachnoid hemorrhage: an observational analytical study
Source: Front Neurol. 2025 Sep 5;16:1601855. doi: 10.3389/fneur.2025.1601855 (PMC12447801; doi:10.3389/fneur.2025.1601855)
Supplement: Supplementary file 1 [file Data_Sheet_1.doc]

Supplemental Table 1 Admission World Federation of Neurological Surgeons Scale scores and serum PTEN-induced putative kinase 1 levels at different time points after aneurysmal subarachnoid hemorrhage

| Time points | Ordinal WFNS scores | | | | | | Discrete WFNS scores | |
| --- | --- | --- | --- | --- | --- | --- | --- | --- |
|  | 1 | 2 | 3 | 4 | 5 | P values | ρ | P values |
| Day 0 | 3.6 (1.8-5.0) | 4.1 (3.7-5.2) | 4.6 (3.7-5.8) | 8.7 (7.2-9.1) | 7.6 (7.4-7.9) | <0.001 | 0.570 | <0.001 |
| Day 1 | 6.9 (4.2-11.2) | 8.0 (5.4-12.2) | 11.6 (5.5-15.9) | 17.6 (14.7-19.7) | 16.7 (16.3-17.1) | <0.001 | 0.581 | <0.001 |
| Day 3 | 8.1 (6.9-16.0) | 11.9 (9.3-17.7) | 17.2 (8.6-21.0) | 30.3 (25.7-34.4) | 41.6 (38.7-44.4) | <0.001 | 0.644 | <0.001 |
| Day 5 | 8.4 (6.4-11.0) | 10.8 (8.6-15.6) | 13.0 (8.0-20.1) | 28.1 (19.6-30.4) | 32.8 (27.5-38.1) | <0.001 | 0.583 | <0.001 |
| Day 7 | 6.0 (4.5-7.0) | 7.9 (6.1-10.5) | 9.6 (5.1-13.1) | 17.7 (12.8-20.6) | 20.5 (19.6-21.4) | <0.001 | 0.586 | <0.001 |
| Day 10 | 3.3 (2.7-4.2) | 4.1 (3.3-6.4) | 5.7 (3.2-7.4) | 10.8 (6.0-13.9) | 14.9 (13.5-16.3) | <0.001 | 0.517 | <0.001 |
| Day 14 | 2.7 (1.9-2.8) | 2.6 (2.0-3.1) | 2.8 (2.1-3.8) | 5.6 (2.5-6.1) | 7.3 (5.8-8.8) | 0.002 | 0.377 | <0.001 |

The Spearman test and Kruskal-Wallis test was applied for correlation analyses and five-group comparisons separately. WFNS stands for World Federation of Neurological Surgeons Scale.

Supplemental Table 2 Admission modified Fisher scale scores and serum PTEN-induced putative kinase 1 levels at several time points following aneurysmal subarachnoid hemorrhage

| Time points | Ordinal mFisher scores | | | | | Discrete mFisher scores | | |  |
| --- | --- | --- | --- | --- | --- | --- | --- | --- | --- |
|  | 1 | 2 | 3 | 4 | P values | | ρ | P values | |
| Day 0 | 3.8 (2.6-5.0) | 4.3 (3.7-5.6) | 7.9 (4.8-8.9) | 7.7 (5.0-8.8) | <0.001 | | 0.493 | <0.001 | |
| Day 1 | 7.4 (5.0-10.8) | 8.6 (5.4-13.3) | 16.3 (12.6-18.9) | 16.7 (10.5-22.9) | <0.001 | | 0.526 | <0.001 | |
| Day 3 | 9.4 (7.4-16.4) | 12.5 (9.7-19.7) | 28.5 (20.7-31.2) | 38.4 (23.3-41.6) | <0.001 | | 0.606 | <0.001 | |
| Day 5 | 9.7 (6.8-12.3) | 11.2 (9.1-18.4) | 20.4 (13.7-29.7) | 27.9 (16.6-33.1) | <0.001 | | 0.517 | <0.001 | |
| Day 7 | 6.5 (4.9-9.1) | 7.9 (6.1-11.2) | 16.5 (10.7-19.4) | 17.0 (8.8-20.5) | <0.001 | | 0.512 | <0.001 | |
| Day 10 | 4.1 (2.4-5.0) | 4.1 (3.3-7.1) | 7.6 (5.3-13.6) | 11.0 (5.9-14.9) | <0.001 | | 0.474 | <0.001 | |
| Day 14 | 2.7 (2.5-2.8) | 2.4 (1.9-3.2) | 4.4 (2.5-5.8) | 5.1 (3.6-7.3) | 0.002 | | 0.329 | 0.002 | |

The Spearman test and Kruskal-Wallis test were employed for completing correlation analyses and four-group comparisons respectively. mFisher indicates modified Fisher scale.

Supplemental Table 3 Admission Glasgow outcome scale scores and serum PTEN-induced putative kinase 1 levels at multiple time points subsequent to aneurysmal subarachnoid hemorrhage

| Time points | Ordinal GOS scores | | | | | | | | | Discrete GOS scores | | |
| --- | --- | --- | --- | --- | --- | --- | --- | --- | --- | --- | --- | --- |
|  | 1 | 2 | 3 | 4 | | 5 | P values | | | ρ | | P values |
| Day 0 | 7.6 (7.3-8.3) | 7.1 (4.4-8.6) | 7.2 (4.8-8.6) | 4.8 (3.8-5.8) | 3.8 (2.2-4.0) | | | <0.001 | -0.552 | | <0.001 | |
| Day 1 | 16.7 (16.2-18.0) | 14.6 (8.9-18.6) | 16.1 (9.5-17.2) | 11.8 (6.1-13.9) | 7.4 (4.6-8.1) | | | <0.001 | -0.559 | | <0.001 | |
| Day 3 | 35.0 (30.5-41.1) | 25.5 (16.5-29.7) | 21.0 (16.3-30.9) | 16.8 (9.2-20.4) | 9.5 (7.5-11.9) | | | <0.001 | -0.599 | | <0.001 | |
| Day 5 | 28.9 (27.5-30.7) | 21.4 (14.6-29.1) | 19.0 (11.4-28.2) | 12.3 (9.1-18.4) | 9.4 (6.8-12.7) | | | <0.001 | -0.519 | | <0.001 | |
| Day 7 | 19.6 (12.8-21.4) | 17.1 (11.1-19.7) | 11.9 (7.9-16.9) | 8.9 (6.5-12.2) | 6.5 (5.1-8.7) | | | <0.001 | -0.477 | | <0.001 | |
| Day 10 | 13.5 (7.1-14.9) | 6.4 (3.3-13.2) | 7.1 (4.9-9.2) | 4.8 (3.4-7.2) | 3.5 (2.5-5.2) | | | 0.001 | -0.430 | | <0.001 | |
| Day 14 | 6.0 (4.3-7.6) | 3.4 (1.8-7.0) | 3.3 (2.5-4.9) | 2.6 (2.0-3.1) | 2.8 (1.9-3.0) | | | 0.021 | -0.302 | | 0.005 | |

The Spearman test and Kruskal-Wallis test were adopted for doing correlation analyses and multiple-group comparisons respectively. GOS signifies Glasgow outcome scale.

Supplemental Table 4 Serum PTEN-induced putative kinase 1 levels at different time points in predicting poor prognosis post-aneurysmal subarachnoid hemorrhage

| Time points | Two-group comparisons | | | ROC curve analyses | |
| --- | --- | --- | --- | --- | --- |
|  | Poor prognosis | Good prognosis | P values | AUC (95% CI) | P values |
| Day 0 | 7.4 (5.0-8.6) | 4.0 (3.6-5.2) | <0.001 | 0.779 (0.670-0.888) | Reference |
| Day 1 | 16.2 (12.3-18.0) | 7.9 (5.3-12.6) | <0.001 | 0.784 (0.677-0.890) | 0.838 |
| Day 3 | 25.7 (20.0-31.2) | 11.9 (8.5-17.9) | <0.001 | 0.799 (0.699-0.898) | 0.602 |
| Day 5 | 20.3 (12.1-29.1) | 10.6 (7.8-15.1) | <0.001 | 0.762 (0.651-0.873) | 0.708 |
| Day 7 | 14.5 (9.1-19.2) | 7.5 (6.0-10.8) | <0.001 | 0.736 (0.622-0.851) | 0.360 |
| Day 10 | 7.2 (4.9-11.9) | 4.1 (3.3-6.4) | 0.001 | 0.706 (0.591-0.821) | 0.131 |
| Day 14 | 4.4 (2.5-5.6) | 2.6 (2.0-3.1) | 0.006 | 0.677 (0.554-0.800) | 0.114 |

The Z test and Mann-Whitney U test was respectively used for comparing areas under curve and serum PTEN-induced putative kinase 1 levels. ROC indicates receiver operating characteristic; AUC, area under curve; 95% CI, 95% confidence interval.

Supplemental Table 5 Serum PTEN-induced putative kinase 1 levels at several time points in forecasting delayed cerebral ischemia subsequent to aneurysmal subarachnoid hemorrhage

| Time points | Two-group comparisons | | | ROC curve analyses | |
| --- | --- | --- | --- | --- | --- |
|  | DCI | Non-DCI | P values | AUC (95% CI) | P values |
| Day 0 | 7.2 (5.3-8.5) | 4.2 (3.6-5.8) | <0.001 | 0.759 (0.653-0.866) | Reference |
| Day 1 | 16.1 (12.9-18.7) | 8.5 (5.3-13.2) | <0.001 | 0.778 (0.672-0.884) | 0.239 |
| Day 3 | 27.6 (19.8-30.5) | 12.4 (8.6-20.7) | <0.001 | 0.788 (0.688-0.888) | 0.475 |
| Day 5 | 19.0 (13.4-30.2) | 11.0 (7.9-19.0) | <0.001 | 0.741 (0.628-0.854) | 0.657 |
| Day 7 | 12.8 (8.9-18.9) | 7.9 (5.9-12.9) | 0.002 | 0.714 (0.597-0.831) | 0.262 |
| Day 10 | 6.8 (4.9-12.3) | 4.7 (3.3-7.2) | 0.011 | 0.676 (0.560-0.792) | 0.071 |
| Day 14 | 3.2 (2.6-5.6) | 2.7 (2.0-3.8) | 0.042 | 0.638 (0.510-0.767) | 0.048 |

The Z test and Mann-Whitney U test was respectively used for comparing areas under curve and biomarker serum levels. ROC indicates receiver operating characteristic; AUC, area under curve; 95% CI, 95% confidence interval; DCI, delayed cerebral ischemia.

**Supplemental Figure Legends**

**Supplemental Figure 1**

Chart showing the study plan regarding aneurysmal subarachnoid hemorrhage.

This observational analytic study entailed cross-sectional assessment and prospective cohort evaluation to determine the dynamic trajectory of serum-based PTEN-induced putative kinase 1 levels and their role as a prognostic biomarker in human aneurysmal subarachnoid hemorrhage.

aSAH indicates aneurysmal subarachnoid hemorrhage; SAH, subarachnoid hemorrhage; PINK1, PTEN-induced putative kinase 1.

**Supplemental Figure 2**

Flow diagram of study design regarding aneurysmal subarachnoid hemorrhage.

This study comprised two parts: the cross-sectional study and the prospective cohort study. The former was designed to discern the evolutionary trajectory of serum PTEN-induced putative kinase 1 levels post-aneurysmal subarachnoid hemorrhage and the latter was performed to ascertain the prognostic influence of serum PTEN-induced putative kinase 1 levels in aneurysmal subarachnoid hemorrhage.

aSAH indicates aneurysmal subarachnoid hemorrhage; DCI, delayed cerebral ischemia; PINK1, PTEN-induced putative kinase 1.

**Supplemental Figure 3**

Serum PTEN-induced putative kinase 1 levels and World Federation of Neurological Surgeons Scale scores following aneurysmal subarachnoid hemorrhage.

Serum PTEN-induced putative kinase 1 levels were demonstrated to be markedly positively correlated with World Federation of Neurological Surgeons Scale scores in aneurysmal subarachnoid hemorrhage (P<0.001).

WFNS denotes World Federation of Neurological Surgeons Scale; PINK1, PTEN-induced putative kinase 1.

**Supplemental Figure 4**

Serum PTEN-induced putative kinase 1 levels among groups with different World Federation of Neurological Surgeons Scale scores after aneurysmal subarachnoid hemorrhage.

Serum PTEN-induced putative kinase 1 levels were significantly elevated in order of World Federation of Neurological Surgeons Scale scores from 1 to 5 in aneurysmal subarachnoid hemorrhage (P<0.001).

WFNS denotes World Federation of Neurological Surgeons Scale; PINK1, PTEN-induced putative kinase 1.

**Supplemental Figure 5**

Serum PTEN-induced putative kinase 1 levels and modified Fisher scores after aneurysmal subarachnoid hemorrhage.

Serum PTEN-induced putative kinase 1 levels were confirmed to be significantly positively related to modified Fisher scores in patients with aneurysmal subarachnoid hemorrhage (P<0.001).

mFisher stands for modified Fisher; PINK1, PTEN-induced putative kinase 1.

**Supplemental Figure 6**

Serum PTEN-induced putative kinase 1 levels across modified Fisher scores post-aneurysmal subarachnoid hemorrhage.

Serum PTEN-induced putative kinase 1 levels were substantially lowest in patients with modified Fisher score 1, followed by the scores 2-3, and highest in those with the score 4 among patients inflicted by aneurysmal subarachnoid hemorrhage (P<0.001).

mFisher signifies modified Fisher; PINK1, PTEN-induced putative kinase 1.

**Supplemental Figure 7**

Serum PTEN-induced putative kinase 1 levels and Glasgow outcome scale scores of patients with aneurysmal subarachnoid hemorrhage.

Serum PTEN-induced putative kinase 1 levels were approved to be notably inversely relevant to Glasgow outcome scale scores in humans diseased of aneurysmal subarachnoid hemorrhage (P<0.001).

GOS indicated Glasgow outcome scale; PINK1, PTEN-induced putative kinase 1.

**Supplemental Figure 8**

Serum PTEN-induced putative kinase 1 levels among patients of distinct Glasgow outcome scale scores following aneurysmal subarachnoid hemorrhage.

Serum PTEN-induced putative kinase 1 levels were evidently in negative proportion to Glasgow outcome scale scores after human aneurysmal subarachnoid hemorrhage (P<0.001).

GOS indicated Glasgow outcome scale; PINK1, PTEN-induced putative kinase 1.

**Supplemental Figure 9**

Serum PTEN-induced putative kinase 1 levels between patients with poor prognosis and those with good prognosis following aneurysmal subarachnoid hemorrhage.

Patients with poor prognosis held significantly higher serum PTEN-induced putative kinase 1 levels than those presenting with good prognosis in humans with aneurysmal subarachnoid hemorrhage (P<0.001).

PINK1 indicates PTEN-induced putative kinase 1.

**Supplemental Figure 10**

Ability of serum-based PTEN-induced putative kinase 1 levels to forecast poor prognosis at 90-day mark subsequent to aneurysmal subarachnoid hemorrhage.

The area under the receiver operating characteristic curve with respect to serum-based PTEN-induced putative kinase 1 levels in predicting a 90-day poor prognosis after subarachnoid hemorrhage was >0.750. The level at 6.6 ng/ml was selected as an optimal value for prognosis anticipation with the maximum Youden index of 0.540.

AUC denotes area under curve; 95% CI, 95% confidence interval.

**Supplemental Figure 11**

Dose-response relationship between serum PTEN-induced putative kinase 1 levels and risk of poor prognosis in aneurysmal subarachnoid hemorrhage.

Using restricted cubic spline analysis, serum PTEN-induced putative kinase 1 levels were linearly associated with the likelihood of poor prognosis following aneurysmal subarachnoid hemorrhage (nonlinear P >0.05).

PINK1 means PTEN-induced putative kinase 1.

**Supplemental Figure 12**

Serum PTEN-induced putative kinase 1 levels between patients with delayed cerebral ischemia and those without such an event following aneurysmal subarachnoid hemorrhage.

Patients with the development of delayed cerebral ischemia verses those without occupied substantially higher serum PTEN-induced putative kinase 1 levels after aneurysmal subarachnoid hemorrhage (P<0.001).

PINK1 indicates PTEN-induced putative kinase 1; DCI, delayed cerebral ischemia.

**Supplemental Figure 13**

Value of serum-based PTEN-induced putative kinase 1 levels for predicting delayed cerebral ischemia following aneurysmal subarachnoid hemorrhage.

The area under the receiver operating characteristic curve for serum-based PTEN-induced putative kinase 1 levels in anticipation of post-SAH delayed cerebral ischemia was 0.755. The level above 6.2 ng/ml distinguished likelihood of delayed cerebral ischemia with the maximum Youden index at 0.470.

AUC stands for area under the curve; 95% CI, 95% confidence interval; DCI, delayed cerebral ischemia.

**Supplemental Figure 14**

Linearity relationship between serum PTEN-induced putative kinase 1 levels and risk of delayed cerebral ischemia subsequent to aneurysmal subarachnoid hemorrhage.

As demonstrated by restricted cubic spline analysis, serum PTEN-induced putative kinase 1 levels were linearly correlated with the possibility of delayed cerebral ischemia following aneurysmal subarachnoid hemorrhage (nonlinear P >0.05).

PINK1 stands for PTEN-induced putative kinase 1.


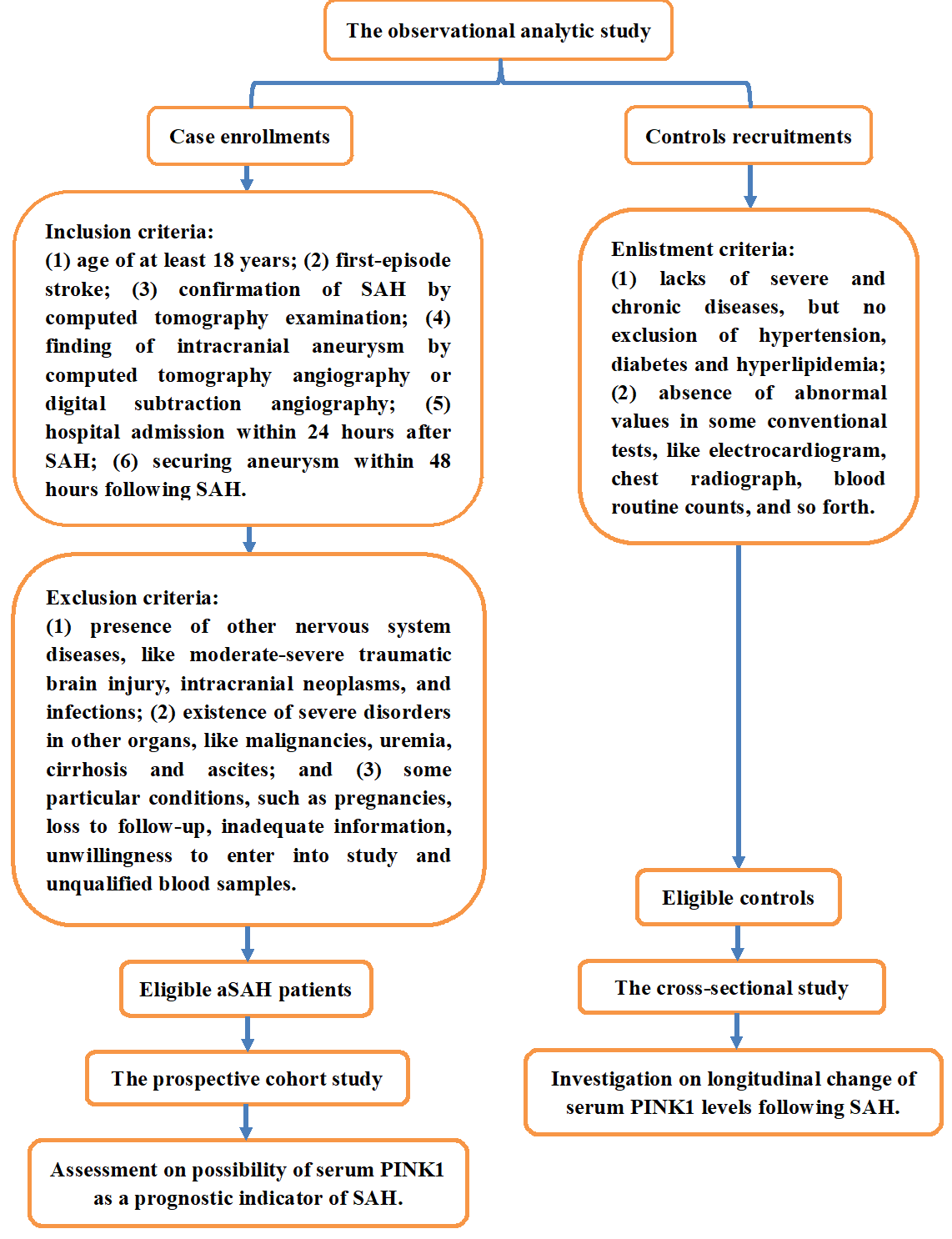


Supplemental Figure 1


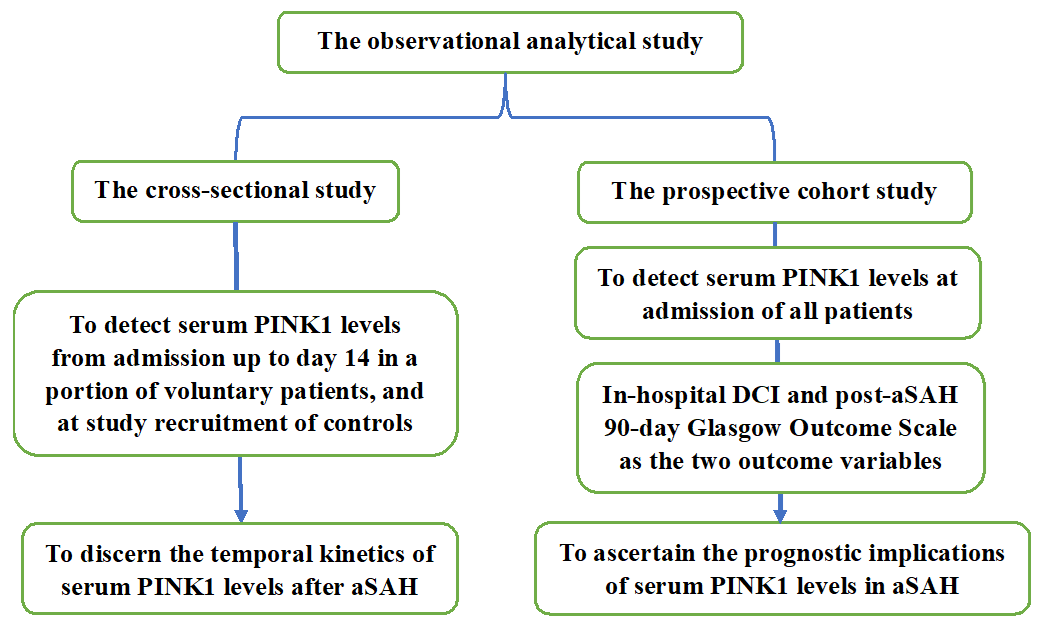


Supplemental Figure 2


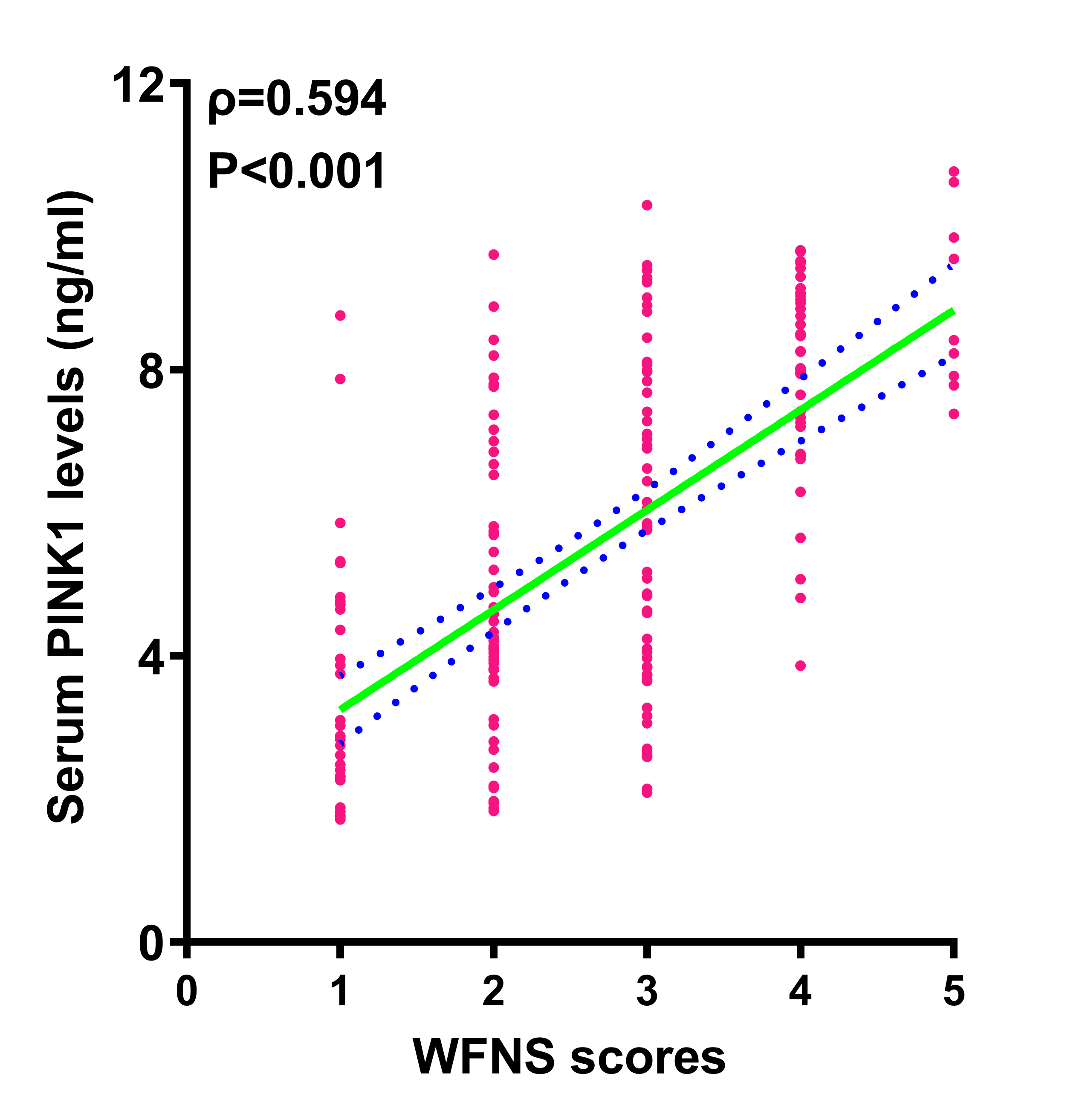


Supplemental Figure 3


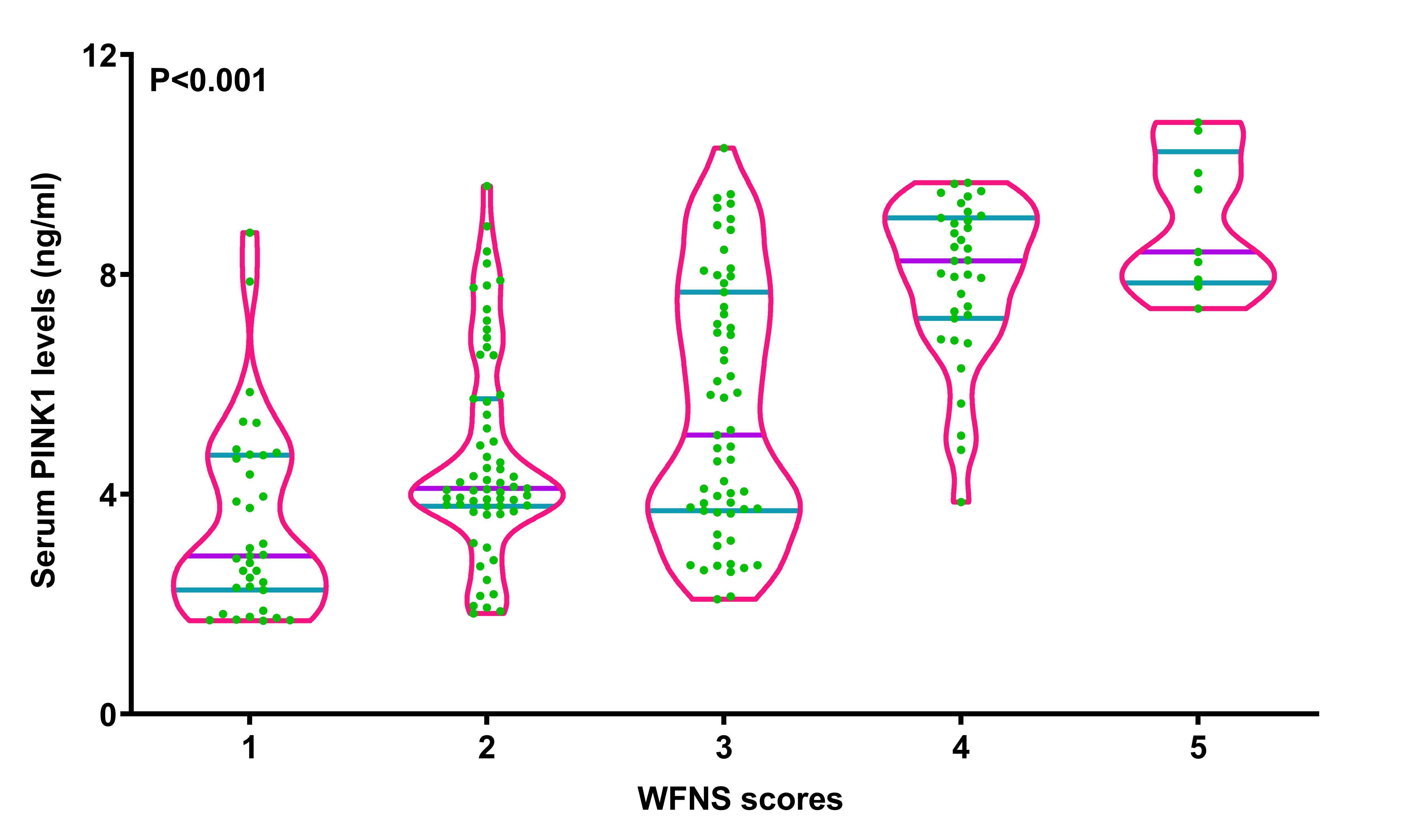


Supplemental Figure 4


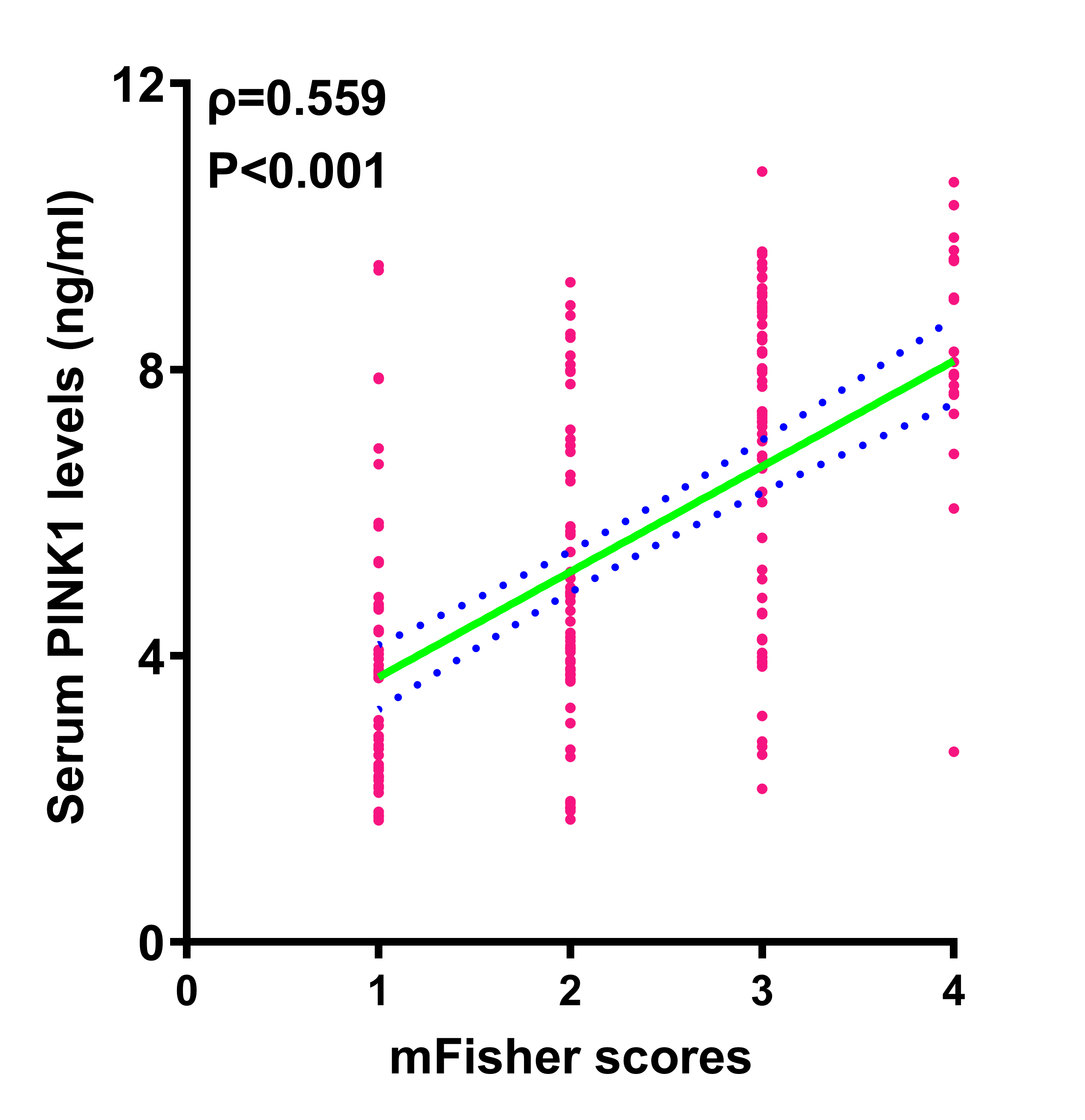


Supplemental Figure 5


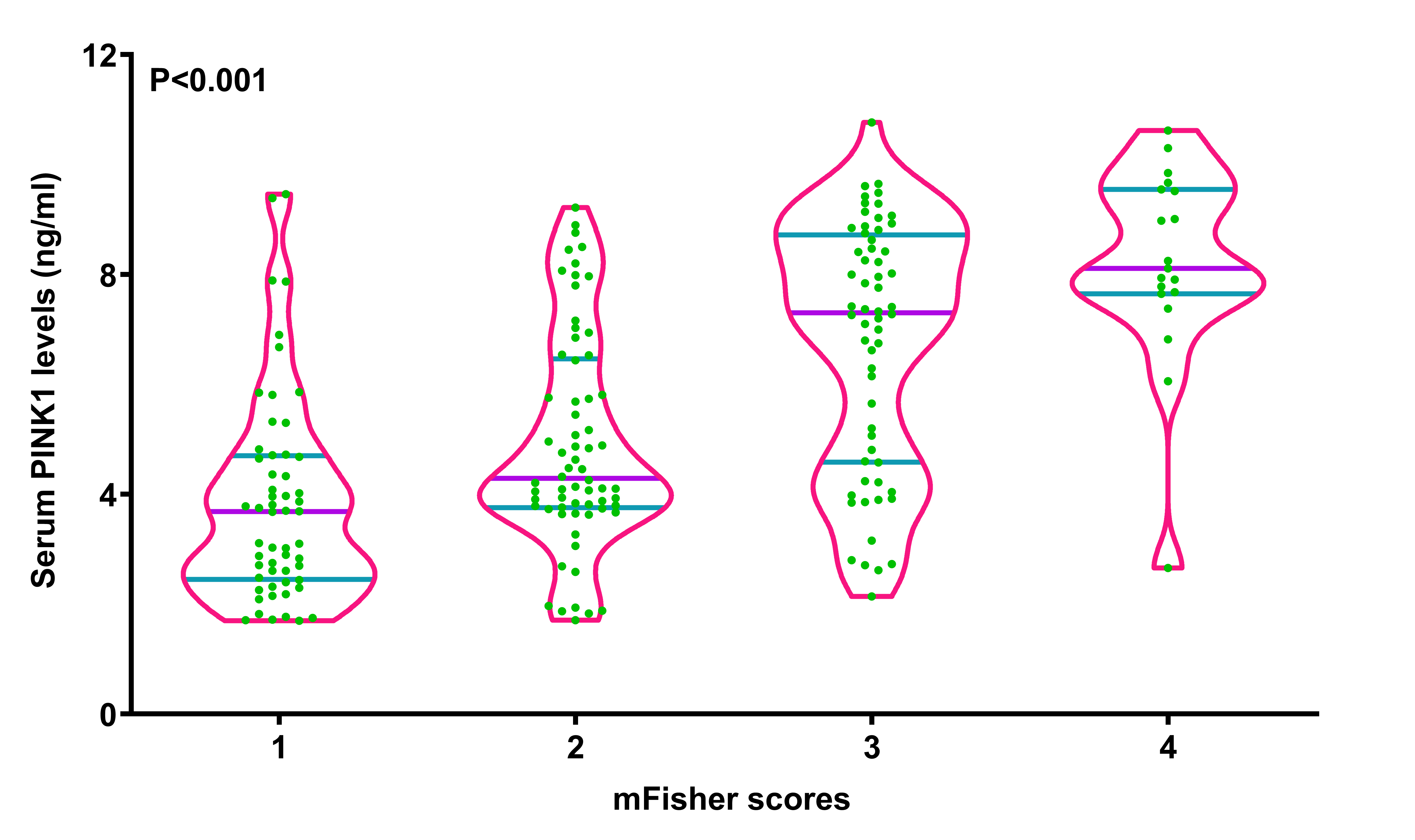


Supplemental Figure 6


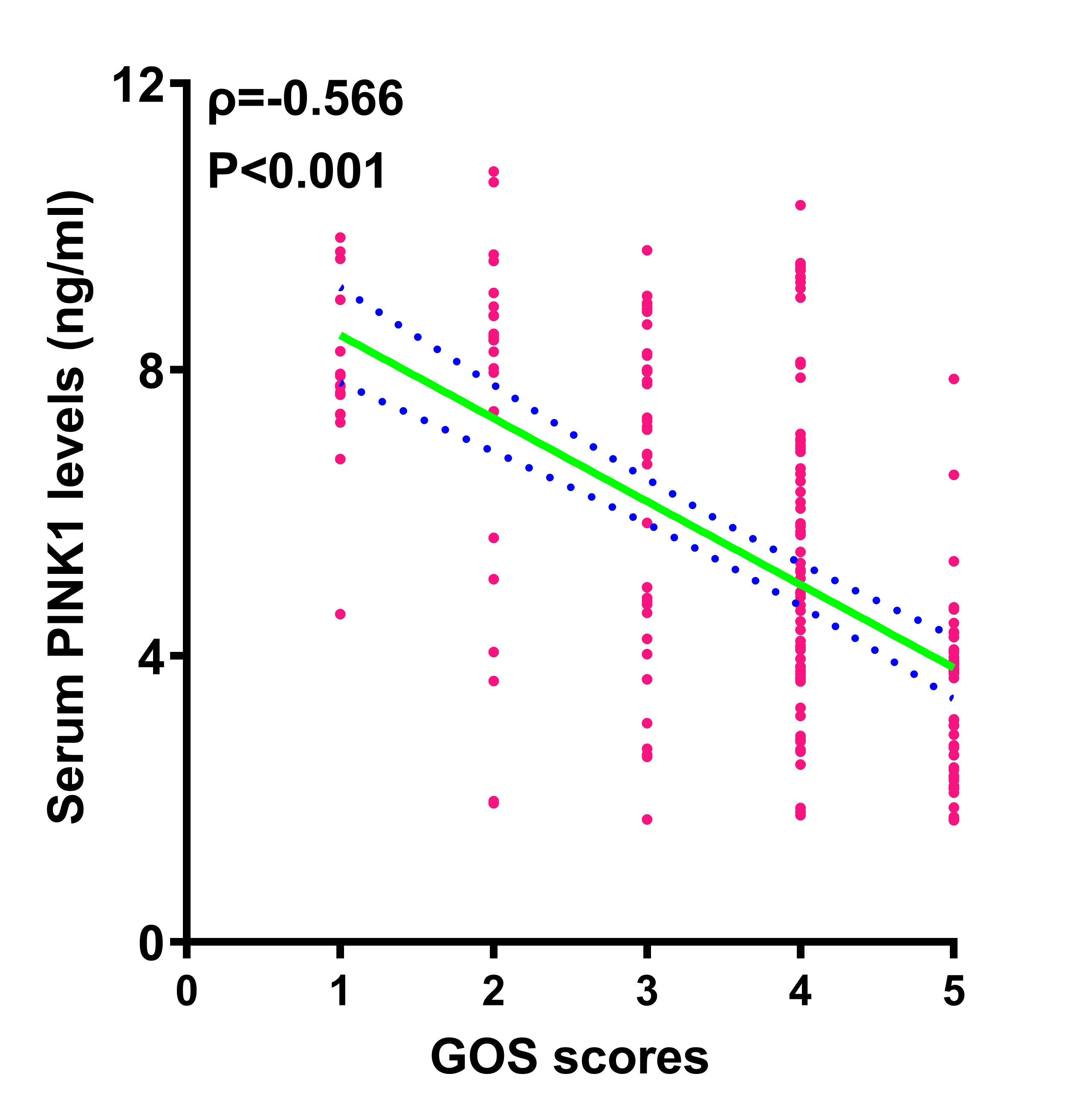


Supplemental Figure 7


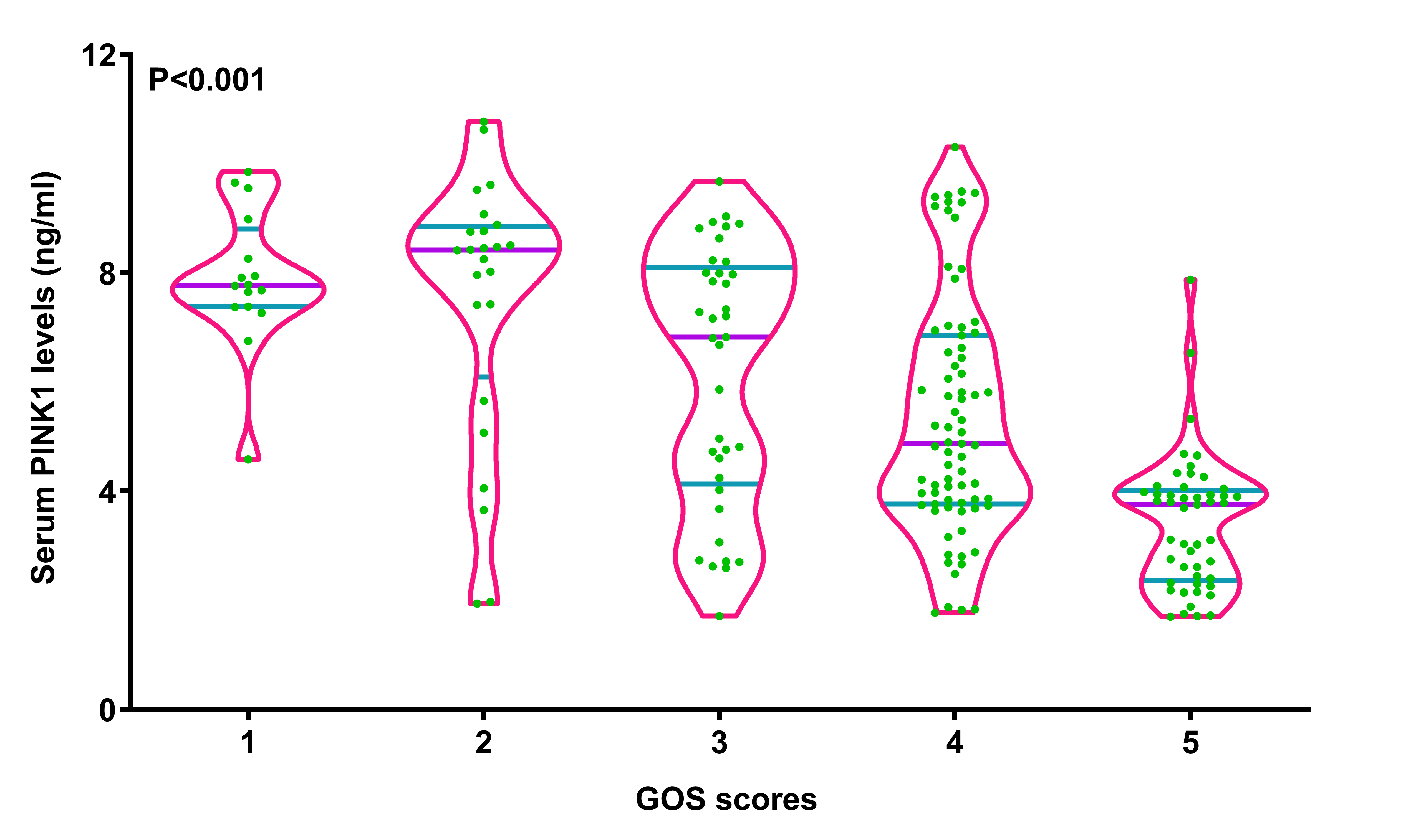


Supplemental Figure 8


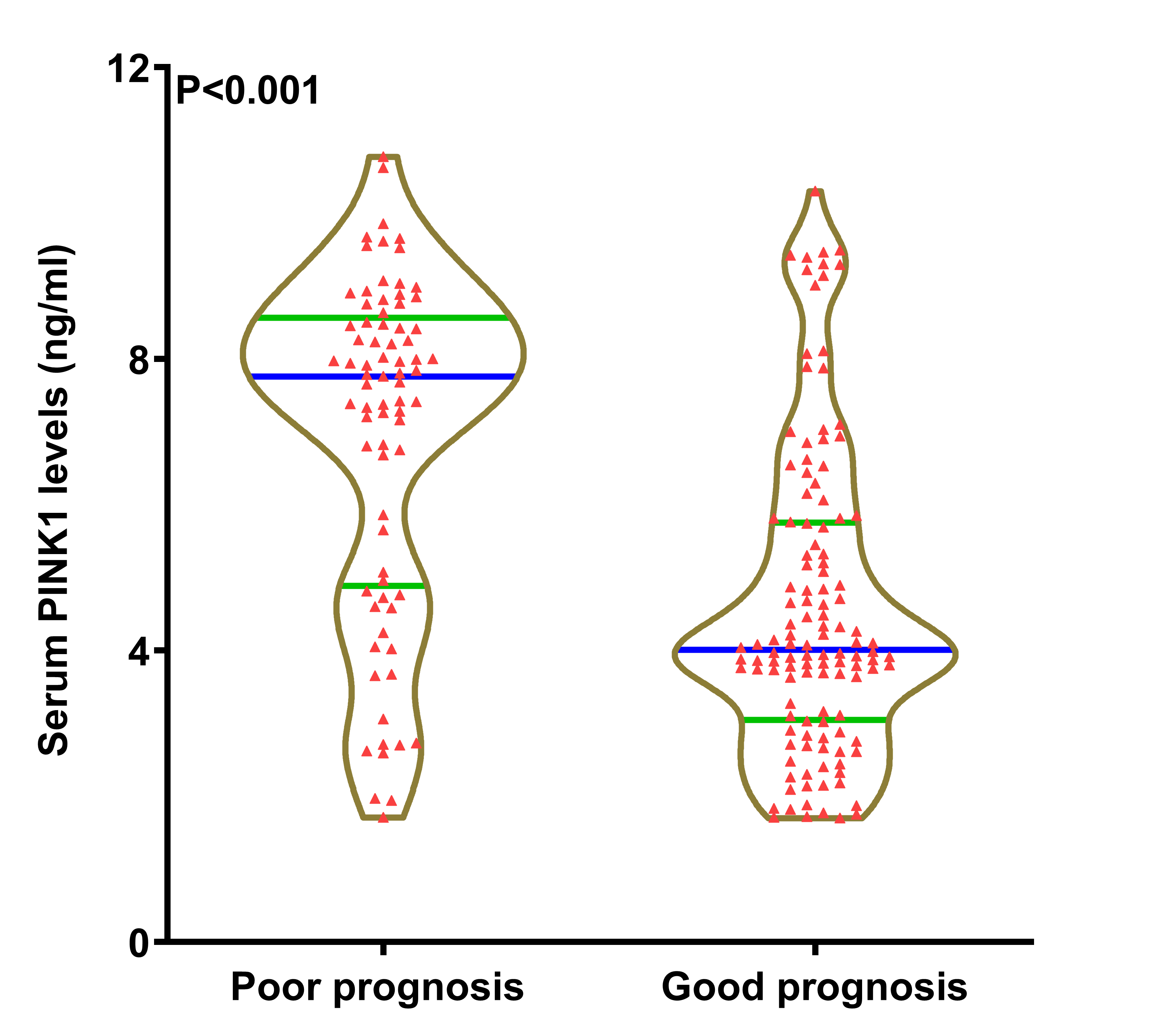


Supplemental Figure 9


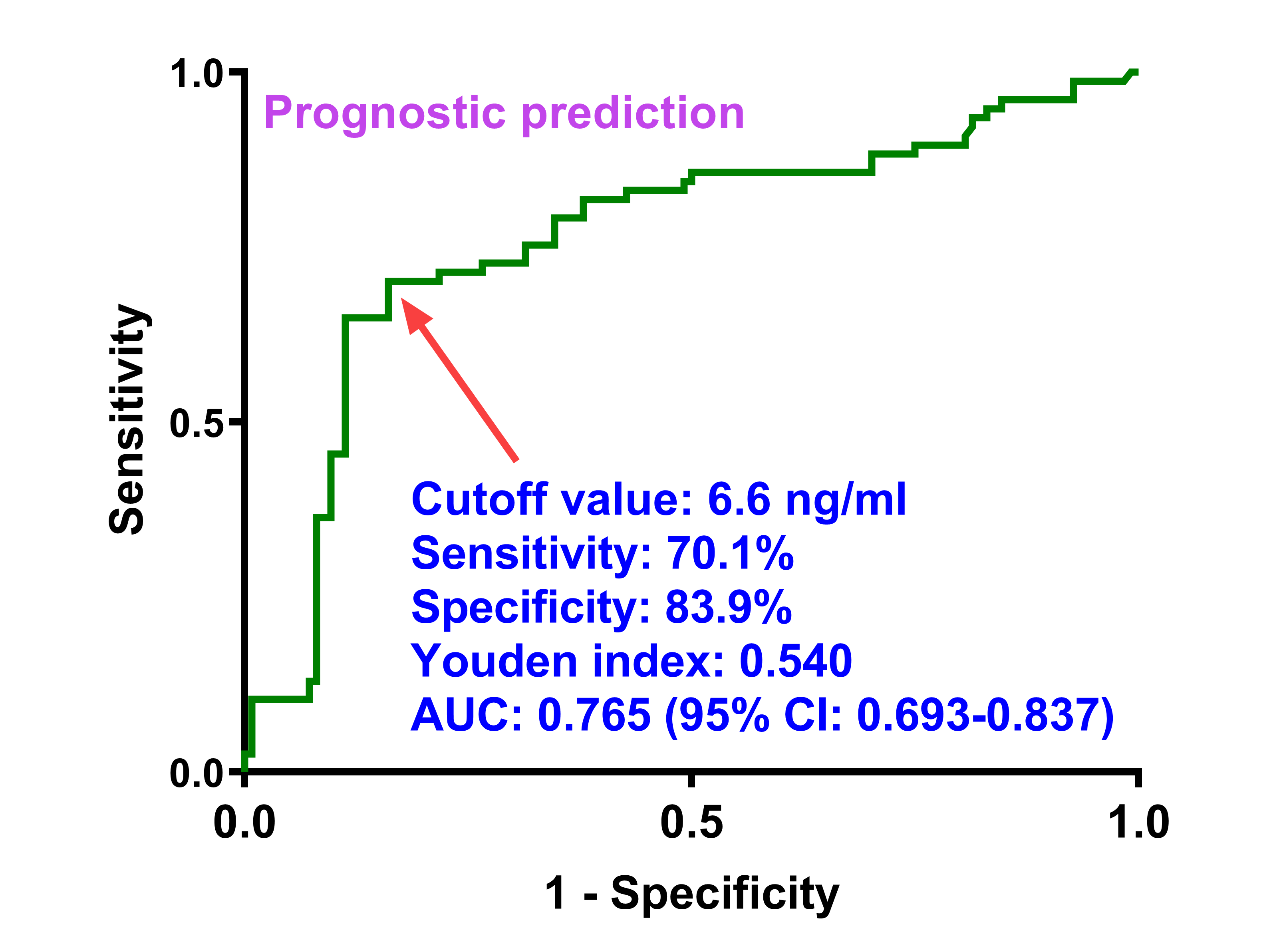


Supplemental Figure 10


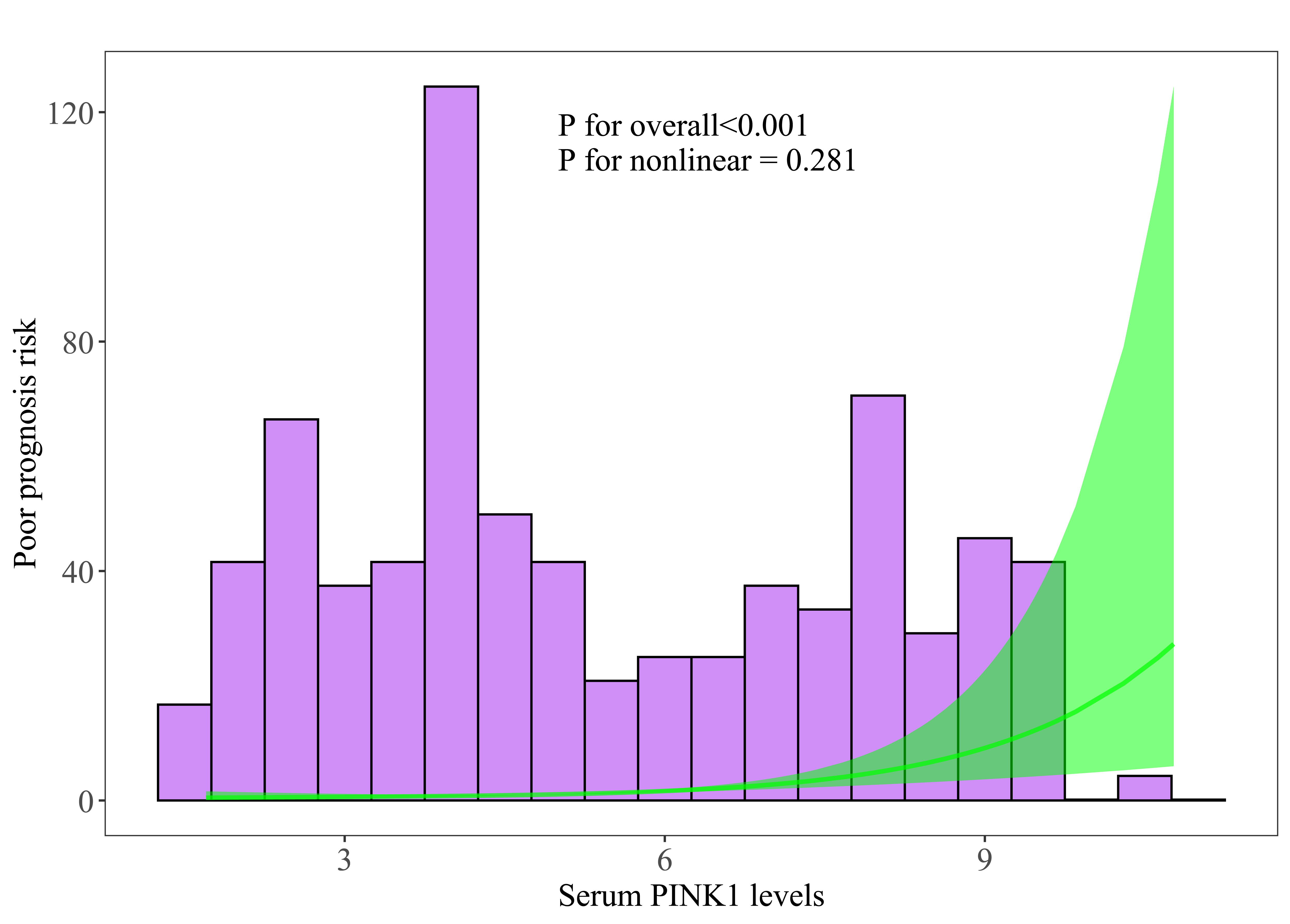
Supplemental Figure 11


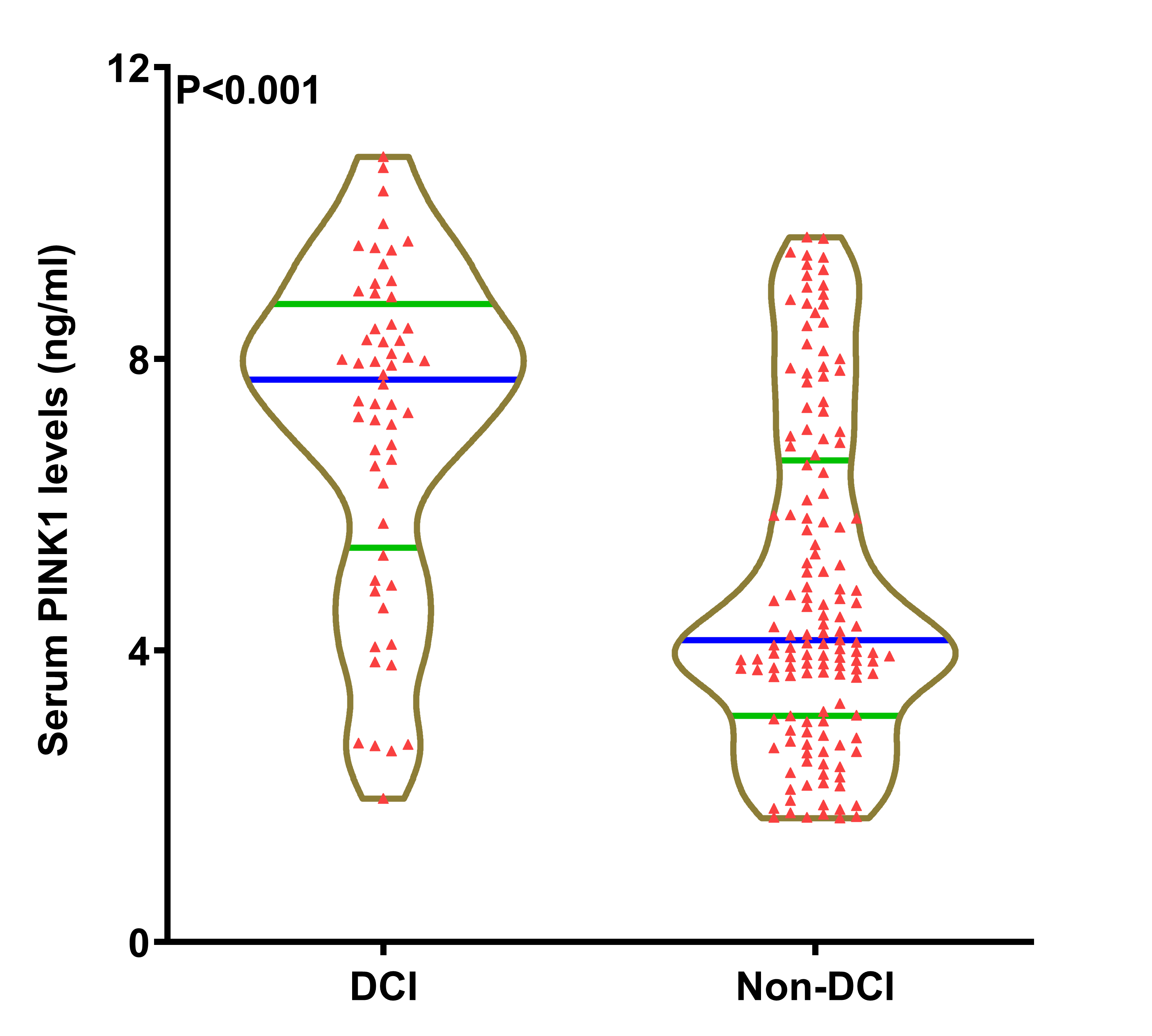


Supplemental Figure 12


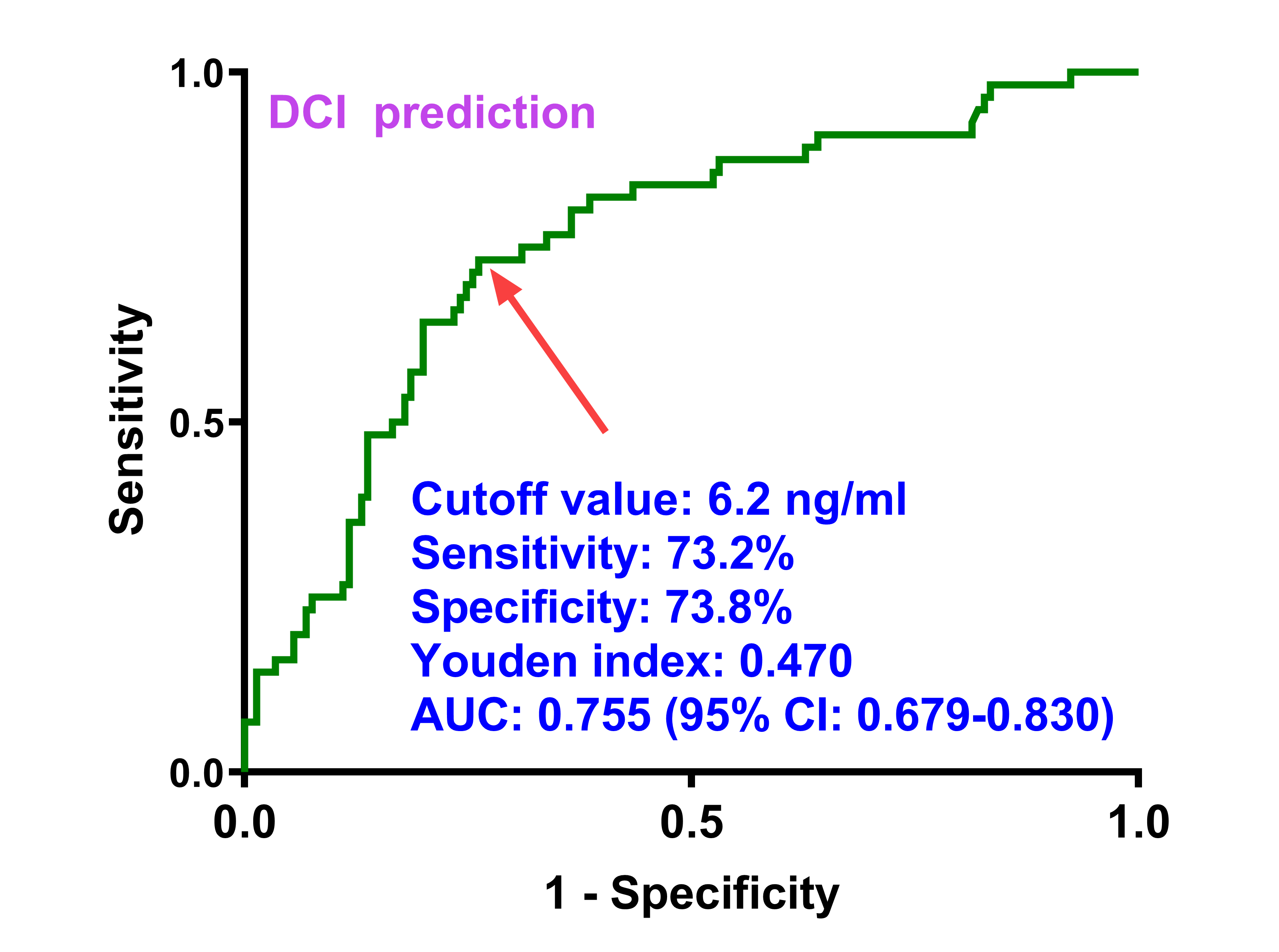


Supplemental Figure 13


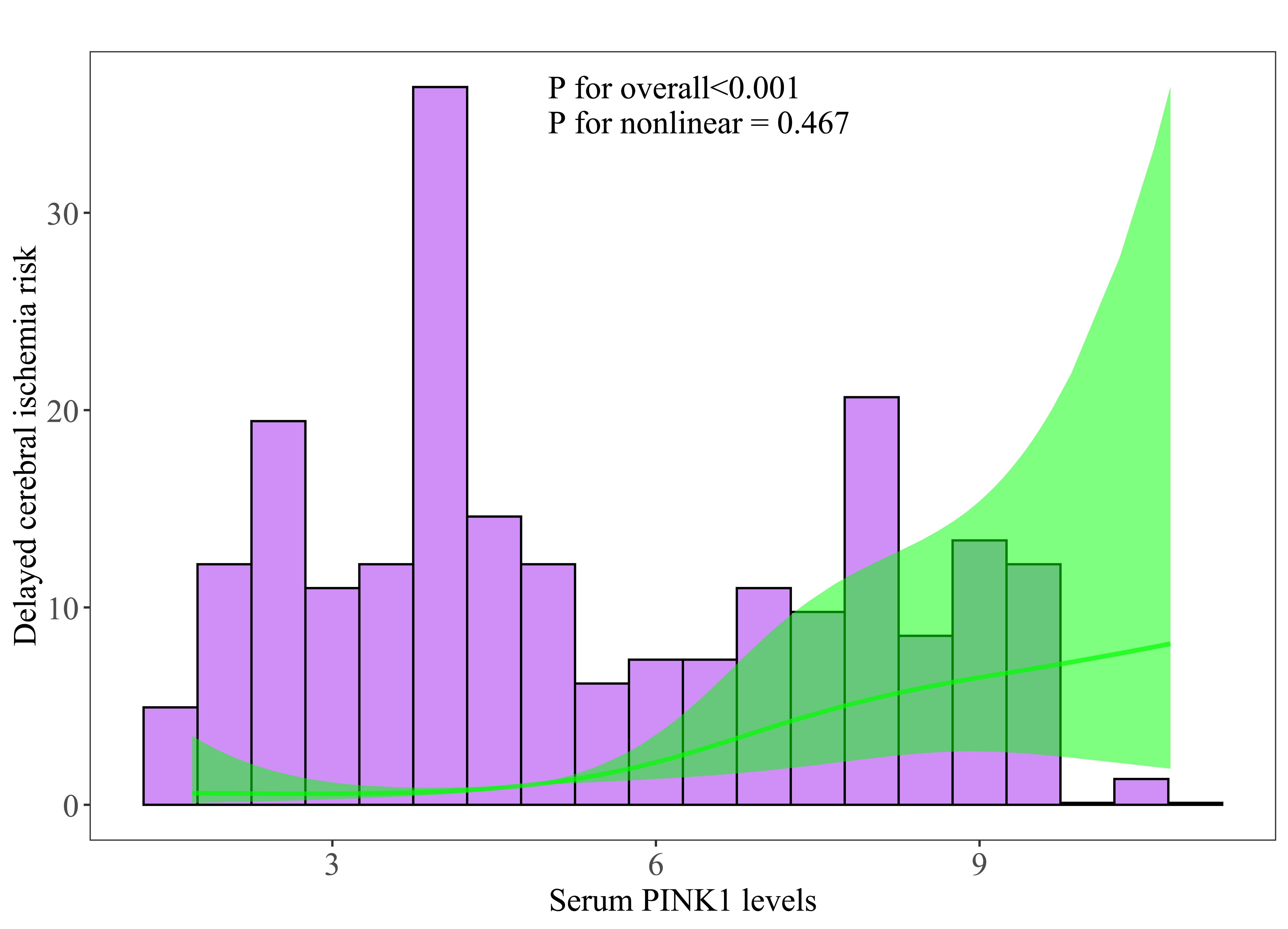


Supplemental Figure 14
